# Lineage tracing studies suggest that the placenta is not a de novo source of hematopoietic stem cells

**DOI:** 10.1371/journal.pbio.3003003

**Published:** 2025-01-28

**Authors:** Xiaowen Chen, Joanna Tober, Martin Dominguez, Alan T. Tang, Jenna Bockman, Jisheng Yang, Sneha Mani, Chin Nien Lee, Mei Chen, Triloshan Thillaikumaran, Patricia Mericko-Ishizuka, Monica Mainigi, Nancy A. Speck, Mark L. Kahn

**Affiliations:** 1 Cardiovascular Institute and Department of Medicine, Perelman School of Medicine, University of Pennsylvania, Philadelphia, Pennsylvania, United States of America; 2 Department of Cell and Developmental Biology, Perelman School of Medicine, University of Pennsylvania, Philadelphia, Pennsylvania, United States of America; 3 Center for Research on Reproduction and Women’s Health, Department of Obstetrics and Gynecology, Perelman School of Medicine, University of Pennsylvania, Philadelphia, Pennsylvania, United States of America; 4 Department of Pathology and Laboratory Medicine, Perelman School of Medicine, University of Pennsylvania, Philadelphia, Pennsylvania, United States of America; The University of Edinburgh School of Biological Sciences, UNITED KINGDOM OF GREAT BRITAIN AND NORTHERN IRELAND

## Abstract

Definitive hematopoietic stem and progenitor cells (HSPCs) arise from a small number of hemogenic endothelial cells (HECs) within the developing embryo. Understanding the origin and ontogeny of HSPCs is of considerable interest and potential therapeutic value. It has been proposed that the murine placenta contains HECs that differentiate into HSPCs. However, during human gestation HSPCs arise in the aorta considerably earlier than when they can first be detected in the placenta, suggesting that the placenta may primarily serve as a niche. We found that the Runx1 transcription factor, which is required to generate HSPCs from HECs, is not expressed by mouse placental ECs. To definitively determine whether the mouse placenta is a site of HSPC emergence, we performed lineage tracing experiments with a Hoxa13^Cre^ allele that specifically labels ECs in the placenta and umbilical cord (UC), but not in the yolk sac or embryo. Immunostaining revealed Hoxa13^Cre^ lineage-traced HECs and HSPCs in the UC, a known site of HECs, but not the placenta. Consistent with these findings, ECs harvested from the E10.5 aorta and UC, but not the placenta, gave rise to hematopoietic cells ex vivo, while colony forming assays using E14.5 fetal liver revealed only 2% of HSPCs arose from Hoxa13-expressing precursors. In contrast, the pan-EC Cdh5-CreER^T2^ allele labeled most HSPCs in the mouse placenta. Lastly, we found that RUNX1 and other HEC genes were not expressed in first-trimester human placenta villous ECs, suggesting that human placenta is not hemogenic. Our findings demonstrate that the placenta functions as a site for expansion of HSPCs that arise within the embryo proper and is not a primary site of HSPC emergence.

## Abstract 2

The placenta has been proposed to harbor hemogenic endothelium that generates hematopoietic stem cells (HSCs). In this study, the authors perform lineage tracing analyses to assess this hypothesis but do not find evidence that the placental vasculature is a site of de novo HSC formation.

## Introduction

The origin of hematopoietic stem and progenitor cells (HSPCs) in the developing embryo has been a subject of intense investigation, in part because understanding where and how HSPCs arise may enable their production for therapeutic purposes. Studies in birds, fish and mammals have established that definitive HSPCs required to drive hematopoiesis throughout the lifetime of an animal arise from RUNX1^ +^ hemogenic endothelial cells (HECs) that appear transiently during embryonic development [1]. Aorta–gonad–mesonephros (AGM) region, embryonic brain vasculature and the placenta have all been reported to generate HSPCs de novo from HECs [2–5]. However, the relative contribution of each of these vascular beds to the final pool of definitive HSPCs remains largely unknown [6].

Studies performed over the past 20 years have demonstrated that the placenta harbors significant numbers of HSPCs [7–10]. In the mouse, HSPCs can be detected in the placenta at embryonic day (E) 11, at about the same time they first appear in the dorsal aorta, and their numbers dramatically expand until E12.5 to E13.5, reaching a similar level as that in the fetal liver before falling in late gestation [5]. Whether the placental vasculature is a site of de novo HSPC formation or it functions primarily for HSPC expansion has been less clear.

The vasculature in the placenta arise from the allantois, an extraembryonic mesodermal tissue distinct from those that give rise to vasculature in the embryo proper and the yolk sac. Ex vivo studies of avian and murine allantois and chick/quail grafting studies in avian embryos have revealed hematopoietic potential in the allantois prior to its fusion with the chorion [11–13]. However, whether the allantois contains HECs that generate hematopoietic progenitors (HPs) in situ is less clear. Vasculogenesis in the allantois occurs prior to its fusion with the chorion, but unlike the yolk sac where vasculogenesis is accompanied by the appearance of primitive erythrocytes, the pre-fusion allantois lacks erythrocytes [14]. The most convincing in vivo study supporting the placenta as a site of de novo HP formation was one that identified HPs in the placenta of Ncx1 mutant mice that lack an ion channel required for normal myocardial contraction [15,16], and therefore presumably lack the blood circulation required to transport HPs from another source to the placenta [17]. However, the inability to generate HSPCs with repopulating activity using E10 mouse placenta culture and the fact that non-placental mammals must achieve the same endpoint without this site suggest that the placenta may instead serve as a supportive niche for HSPC expansion in a role parallel to that of the fetal liver or the caudal hematopoietic tissue in zebrafish [18–20]. Analysis of the human placenta has also shed doubt on its role as a site of de novo HSPC generation. In contrast to the mouse, which has a very short gestation time during which definitive HSPCs can be found at multiple sites almost concurrently, the timing of human HSPC development is temporally better resolved. During human gestation, HSPC emergence in the AGM region has been shown to take place at least 5 days earlier than the appearance of significant HSPCs in the placenta, suggesting that HSPCs from the AGM colonize the placenta [21]. The exact source of human placenta HSPCs is still debated [22].

Determining the developmental origin of HSPCs remains a challenging task, as HSPCs are mobile cells that are redistributed across numerous vascular beds [19]. Genetic lineage tracing has been successfully used to advance our understanding of the ontogeny of murine HSPCs and their niches [23,24], e.g., demonstrating that definitive HSPCs are generated from vascular ECs through endothelial-to-hematopoietic transition (EHT) [25]. In the present study, we utilize a recently described Hoxa13-Cre driver with specific expression in the allantois (Hoxa13^Cre^) [26] to rigorously determine whether placental HSPCs arise from allantoic precursors, including placental HECs. Our results demonstrate that very few if any HSPCs are generated de novo in the placenta.

## Results

### Proliferative RUNX^ + ^c-Kit^ +^ HSPCs are located within the vascular lumen of mouse placental vessels during mid-gestation

**Fig 1:**
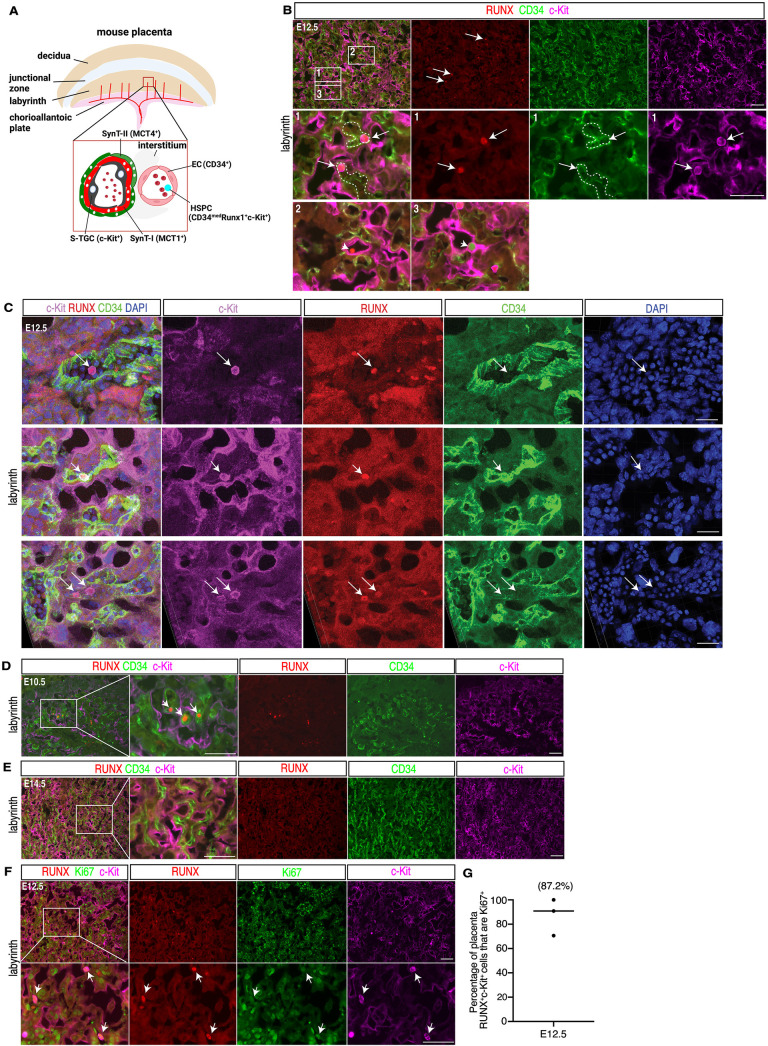
Identification of RUNX^ + ^c-Kit^ +^ HSPCs in the mouse placental labyrinth. **(A)** Schematic diagrams of the mouse E12.5 placenta (top) and maternal-fetal vascular arrangement within the labyrinth (bottom) are shown. Red lines indicate fetal blood vessels. EC, fetal endothelial cells; HSPC, hematopoietic stem and progenitor cells; S-TGC, sinusoidal-trophoblast giant cells; SynT-1, syncytiocytotrophoblast layer I; SynT-2, syncytiocytotrophoblast layer II. **(B)** Detection of CD34^ +^ HSPCs and ECs (green), RUNX^ +^ HSPCs (red) and c-Kit^ +^ HSPCs and trophoblasts (magenta) in wild-type E12.5 placenta (*N* = 3). White arrows indicate HSPCs. 1, 2 and 3 indicate boxed regions shown below. White dotted lines outline fetal blood vessels. **(C)** 3D snapshot view of immunostaining for c-Kit (magenta), RUNX (red), CD34 (green) and DAPI (blue) on 20 μm frozen section of E12.5 mouse placenta. White arrows indicate placenta HSPCs. **(D)** Immunofluorescence staining for CD34 (green), RUNX (red) and c-Kit (magenta) on wild-type E10.5 placenta sections (*N* = 3). The boxed region is shown at higher magnification in the panels to the right. White arrows indicate RUNX^ +^ fetal hematopoietic cells. **(E)** Detection of CD34^ +^ HSPCs and ECs (green), RUNX^ +^ HSPCs (red) and c-Kit^ +^ HSPCs and trophoblasts (magenta) in wild-type E14.5 placenta (*N* = 3). Boxed region is shown at higher magnification in the panels on the right. No RUNX^ + ^c-Kit^ +^ HSPCs are visible. **(F)** Immunofluorescence staining for Ki67 (green), RUNX (red) and c-Kit (magenta) on wild-type E12.5 placenta (*N* = 3). Lower panels showed the boxed region in the upper left panel. White arrows indicate placenta HSPCs. **(G)** The percentage of Ki67^ +^ placental RUNX^ + ^c-Kit^ +^ cells at E12.5. All scale bars =  100 μm, except 20 μm in C. The data underlying this figure can be found in S1 Data.

To spatially identify HSPCs in the intact placenta, we performed immunostaining for RUNX, c-Kit and CD34, established marker genes for these cells. c-Kit was strongly expressed in cells within the lumen of fetal blood vessels in the E12.5 placenta labyrinth (Fig 1A, B), and in MCT1^ +^ sinusoidal-trophoblast giant cells (S-TGCs) that line the maternal blood vascular space (S1A, B Fig). Circulating RUNX^ + ^c-Kit^ +^ hematopoietic cells were identified in the lumen of CD34^ +^ endothelial-lined fetal vessels in the placenta, but not within the lumen of c-Kit^ +^ trophoblast-lined vessels that carry maternal blood (Fig 1B). Confocal analysis of immunostained thick sections occasionally revealed free circulating HSPCs in larger caliber vessels within the labyrinth (e.g., upper panel in Fig 1C), but most HSPCs were noted to be in close physical contact with labyrinth ECs (middle and lower panels in Fig 1C). This arrangement is similar to that observed for HSPCs in the zebrafish CHT and mouse fetal liver [20], where ECs serve a niche function for HSPC expansion. RUNX^ + ^c-Kit^−^ fetal hematopoietic cells were also detected in the labyrinth region of the E10.5 placenta (Fig 1D). We did not observe RUNX^ + ^c-Kit^ +^ cells in the labyrinth at E14.5 (Fig 1E) in agreement with prior studies reporting that most HSPCs have migrated out of the placenta by that stage [20]. To assess whether the placenta is a site of HSPC expansion, we co-stained E12.5 placenta sections with antibodies recognizing the proliferative marker Ki67, RUNX and c-Kit (Fig 1F). We observed that 87.2% of RUNX^ + ^c-Kit^ +^ cells in E12.5 placenta were Ki67^ +^ (Fig 1G), indicating that this cell population is highly proliferative. Staining for CD43, a marker of pre-HSCs and multi-potent progenitors that are known to be highly proliferative [27–29], revealed that the fraction of Runx1^ +^ cells that co-express CD43 rises from < 20% at E11.5 to > 80% at E12.5, the time point of peak proliferative rate in placental RUNX^ +^ cells (S2 Fig). These findings show that RUNX^ + ^c-Kit^ +^ cells in the placental vasculature are luminal, often associated with placental ECs, and actively proliferating with pre-HSC marker expression.

### Mouse placental ECs do not express the endothelial hemogenic transcription factor RUNX1 or other RUNX proteins

**Fig 2:**
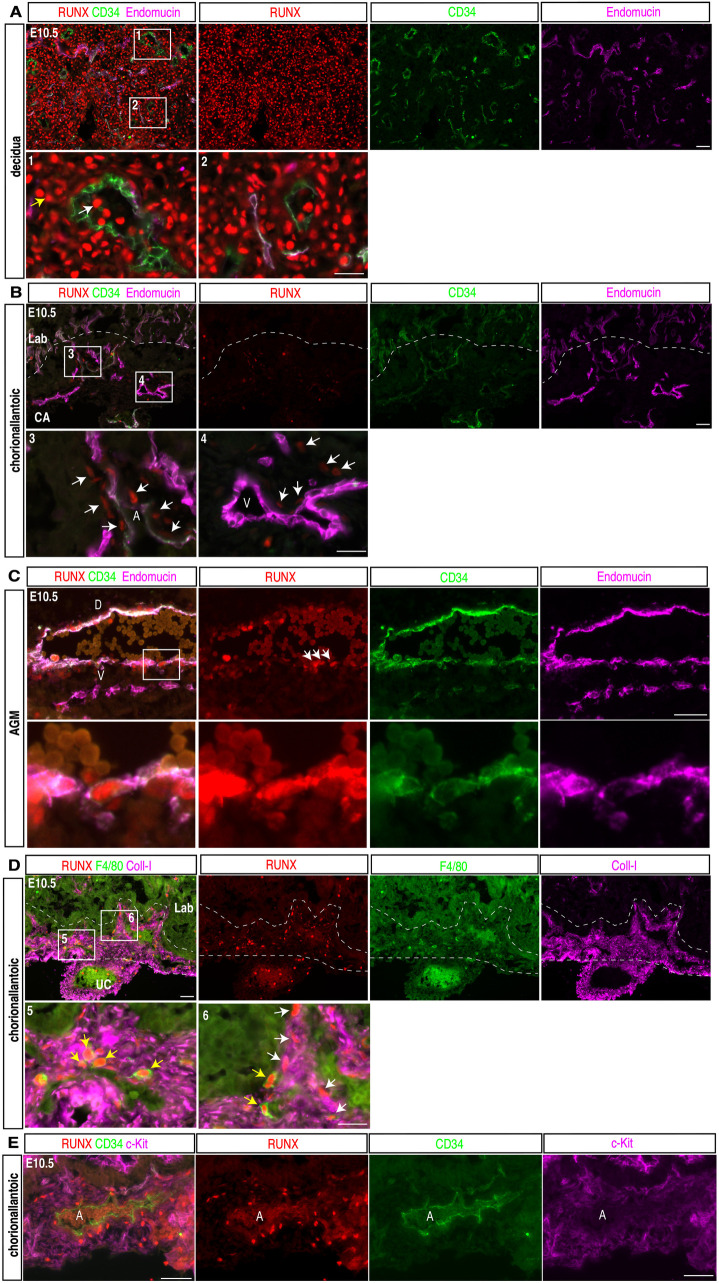
RUNX proteins are not expressed in mouse placental ECs. (**A**) Immunofluorescence staining for RUNX (red), CD34 (green) and Endomucin (magenta) in the maternal decidua of E10.5 mouse placenta sections (*N* = 3 placentas). Lower images indicate boxed regions 1 and 2. White arrows indicate RUNX^ +^ hematopoietic cells in maternal vessels. Yellow arrows indicate RUNX^ +^ stromal cells within the decidua. (**B**) Immunofluorescence staining for RUNX (red), CD34 (green) and Endomucin (magenta) in the fetal labyrinth (Lab) and chorioallantoic (CA) plate of the E10.5 mouse placenta (*N* = 3 placentas). Lower images show boxed regions 3 and 4. Dotted line indicates the border between the CA and labyrinth regions. White arrows indicate RUNX^ +^ stromal cells that are adjacent to Endomucin^ +^ ECs. The images in panels A and B were obtained using identical imaging settings. (**C**) Immunofluorescence staining for RUNX (red), CD34 (green) and Endomucin (magenta) in the E10.5 AGM region (*N* = 3 placentas). Lower images show boxed regions in the upper panel. White arrows indicate RUNX^ +^ hemogenic ECs. D and V indicate dorsal and ventral side of the vessel. (**D**) Immunofluorescence staining for RUNX (red), F4/80 (green) and Coll-I (magenta) in E10.5 mouse placenta sections (*N* = 3 placentas). Dotted lines outline the CA region between the umbilical cord (UC) and labyrinth (Lab). Lower images show boxed regions 5 and 6. White arrows indicate RUNX^+^Coll-I^ +^ stromal cells; yellow arrows indicate RUNX^ + ^F4/80^ +^ fetal macrophages. (**E**) Immunofluorescence staining for RUNX (red), CD34 (green) and c-Kit (magenta) at the CA plate of the E10.5 wild-type mouse placenta (*N* = 3 placentas). A, artery. Scale bars: 20 μm.

The Runx1 transcription factor is expressed in HECs at known sites of HSPC emergence such as the AGM and has been shown to be necessary for EHT in vivo [30]. Previous studies using a Runx1-LacZ allele reported lacZ activity in allantoic mesenchymal cells and placental ECs [11,17], findings consistent with hemogenic placental endothelium. However, direct measurement of Runx1 protein expression in the placenta vasculature has not been reported. To directly assess the expression of all RUNX proteins (RUNX1–3), we performed immunostaining of the E10.5 mouse placenta using an antibody that recognizes all three proteins. Strong nuclear RUNX protein staining was observed in most decidual stromal cells (Fig 2A), consistent with previous studies implicating Runx1 in mouse decidualization [31,32]. RUNX proteins were also expressed by some maternal circulating blood cells in decidual vessels lined by CD34^ +^ endothelium, but not in decidual ECs (Fig 2A, insets 1 and 2). RUNX proteins were detected at low levels in a small number of extra-luminal, Endomucin-negative cells in the mesenchyme of the fetal chorioallantoic (CA) region of the placenta (Fig 2B). We did not observe any Endomucin^ +^ placental ECs that were RUNX^ +^ (Fig 2B) among 800 ECs in three sections each from two placentas. We detected RUNX staining in cells in the perivascular mesenchyme in the CA region, but not in any Endomucin^ +^ ECs of either the larger Endomucin^low^ arteries or Endomucin^high^ veins (0 out of 150 ECs in four sections of two placentas) (Fig 2B, inset panels 3 and 4). In contrast, we detected strong nuclear RUNX staining in both CD34^ +^ ECs and sub-aortic mesenchymal cells in the AGM of the same animals (Fig 2C). Additional immunostaining revealed that most RUNX^ +^ cells in the CA region were either F4/80^ +^ placental fetal macrophages or Collagen type I positive (Coll-I) stromal cells (Fig 2D). Whole mount staining of 150 micrometer thick E11.5 placenta sections also revealed strong RUNX expression in decidual cells and hematopoietic cells, weak RUNX expression in the perivascular space of the CA region, and none in any placenta ECs (S3A, B Fig). Consistent with these histologic findings, analysis of the publically available single nuclei RNA-seq database of the mouse E9.5, E10.5, E12.5 and E14.5 placenta [33] revealed little or no expression of Runx1 in placental ECs at all four time points (S4A Fig). In contrast, this study revealed robust Runx1 expression in hematopoietic and decidual stromal cells (S4A Fig), consistent with our immunostaining data (Fig 2A). We also found Runx1 was the strongest expressed of the three Runx genes (S4C Fig).

A histologic hallmark of EHT is the emergence of c-Kit^ +^ hematopoietic clusters from hemogenic endothelium at sites such as the AGM [25]. We therefore next performed immunostaining to search for similar clusters in the large vessels of the placenta. We failed to detect any RUNX^ + ^c-Kit^ +^ cells that were closely attached to a CD34^ +^ artery and resembled clusters in thin sections (Fig 2E) or thick sections (S3A, B Fig). We next assessed expression of Sox17 and Gata2, transcription factors known to participate in HSPC development [34,35], in the placenta. Robust Gata2 expression was detected in trophoblast cells and their progenitors, consistent with its known role in regulating trophoblast development [36], but not in placental ECs (S4D Fig). Sox17 was strongly expressed in the Crypt of Duval, a structure derived from endodermal cells, as well as in arterial ECs of the placenta (S4D Fig), consistent with a known role for Sox17 in maintaining arterial integrity [37]. Immunostaining for RUNX was highly concordant with analysis of Runx1 gene expression in placental single nuclei RNA-seq data (S4A, B Fig). Finally, we evaluated expression of other known hemogenic EC genes, *Gfi1* and *Hey2*, by RNAScope assay and single nuclei RNA-seq [38,39]. There was no expression of *Gfi1* in placenta ECs (S5B Fig) while AGM endothelium showed high *Gfi1* expression (S5A Fig). These data were again highly concordant with those in the single nuclei RNA-seq database (S5C, D Fig). Thus, placental endothelium does not exhibit the gene expression signature associated with well-defined hemogenic endothelium such as that in the AGM, and fails to exhibit morphologic features such as cluster formation that are characteristic of EHT.

### RUNX^ + ^c-Kit^ +^ HSPCs in the mouse placenta are not lineage-traced by Hoxa13^Cre^

**Fig 3:**
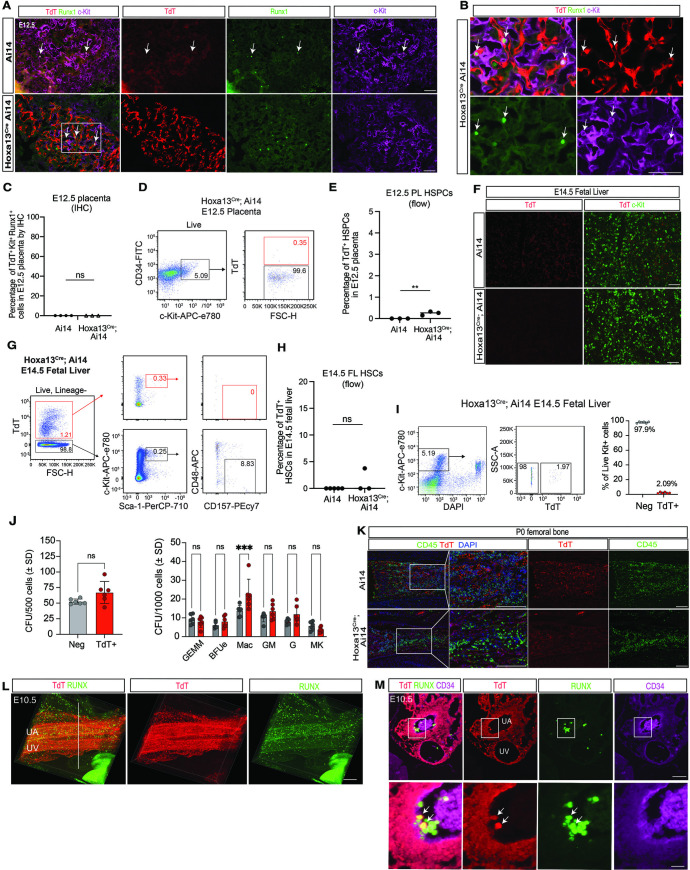
Mouse placental HSPCs are not lineage traced by Hoxa13^Cre^. (**A**) Immunofluorescence staining for RUNX (green), TdT (red) and c-Kit (magenta) in E13.5 Ai14 only (upper) and Hoxa13^Cre^; Ai14 (lower) placenta (*N* = 3 placentas). White arrows indicate RUNX^ + ^c-Kit^ +^ cells. (**B**) Boxed region shown in (A) at higher magnification. (**C**) Quantification of TdT^ +^ placental RUNX^ + ^c-Kit^ +^ cells in Ai14 and Hoxa13^Cre^; Ai14 placentas (*N* = 3 placentas). ns, not significant. (**D**) Flow cytometric detection of TdT^ +^ CD34^med^c-Kit^ +^ cells in E12.5 placentas is shown. Red numbers indicate the percentage of TdT^ +^ cells in the gated population. (**E**) Quantification of percentage of TdT^ +^ cells in the CD34^med^c-Kit^ +^ HSPC population from the E12.5 placenta. (**F**) Immunofluorescence staining for c-Kit (green) and TdT (red) on E14.5 Ai14 only (upper) and Hoxa13^Cre^; Ai14 (lower) fetal liver sections. (**G**) Flow cytometric analysis of Lin^−^c-Kit^+^Sca1^ + ^CD48^−^CD150^ +^ phenotypic LT-HSCs in the E14.5 fetal liver of Hoxa13^Cre^; Ai14 animals. Red numbers and gates indicate percentage of TdT^ +^ cells; black numbers and gates are TdT^−^ cells. (**H**) Quantification of TdT^ +^ Lin^−^c-Kit^+^Sca1^ + ^CD48^−^CD150^ +^ phenotypic LT-HSCs in the E14.5 fetal liver (FL) of Ai14 and Hoxa13^Cre^; Ai14 animals (*n* = 5 for Ai14 control and *n* = 3 for Hoxa13^Cre^; Ai14. (**I**) FACS plot and percentage of TdT^ + ^c-Kit^ +^ HSPCs in E14.5 fetal liver of Ai14 and Hoxa13^Cre^; Ai14 animals. (**J**) Methylcellulose colony forming unit (CFU) assays show there are no differences in the frequencies of CFUs in TdT^−^c-Kit^ +^ and TdT^ + ^c-Kit^ +^ progenitors (mean ± SD, *n* = 6). Graph on the left shows all CFUs and graph on the right are specific progenitors. GEMM, granulocyte-erythrocyte, monocyte-megakaryocyte progenitors; BFUe, burst forming unit erythroid progenitors; Mac, macrophage progenitors; GM, granulocyte-monocyte progenitors; G, granulocyte progenitors; MK, megakaryocyte progenitors. (**K**) Immunofluorescence staining for CD45 (green), TdT (red) and DAPI (blue) on P0 femur sections of Ai14 and Hoxa13^Cre^; Ai14 animals. (**L**) 3D Snapshot view of Hoxa13^Cre^; Ai14 E10.5 UCs stained for RUNX (green) and TdT (red) (*N* = 3 UCs). (**M**) Representative image showing immunostaining of E10.5 Hoxa13^Cre^; Ai14 umbilical cord section for TdT (red), RUNX (green) and CD34 (magenta) (three sections per cord for *N* = 3 umbilical cords). UA, umbilical artery; UV, umbilical vein. Lower panel shows the enlarged area in the white box from the upper panel. White arrows indicate TdT^ +^ hematopoietic cells with the hematopoietic cluster. Scale bar: 100 um. Scale bars: 100 μm (A), 50 μm (B, F, L, K and M). The data underlying this figure can be found in S1 Data.

We recently generated a Hoxa13^Cre^ allele that is active in the early allantois and the placental vascular endothelial precursors that arise there [26]. *Hoxa13* is transiently expressed in the allantois and allantois-derived ECs from E7.5 to E9.5 [40]. Lineage tracing studies demonstrated that the Hoxa13^Cre^ allele labels virtually all ECs in the placenta and some ECs in the umbilical vessels but does not label ECs within the yolk sac or embryo proper, including those at hemogenic sites such as the AGM or cerebral vessels [26] (S6A, B Fig). Consistent with 100% EC labeling, we observed the complete loss of placental ECs in Hoxa13^Cre^; R26-LSL-DTA animals in which the diphtheria toxin fragment A (DTA) is expressed in all Hoxa13 lineage cells (S6C Fig). Examination of *Hoxa13* gene expression in HSPCs throughout HSPC ontogeny [41] revealed no detectable expression level at any stage or site of hematopoiesis (S6D Fig). In contrast, Hoxa9, a related homeobox gene that has been shown to play an important role in hematopoiesis [42], was readily detected at all stages and sites (S6E Fig). Thus, Hoxa13^Cre^ is an appropriate tool with which to identify hematopoietic cells that originate from placental ECs via EHT. To determine if placental ECs give rise to HSPCs, we crossed Hoxa13^Cre^ with the sensitive Ai14 Cre reporter allele (R26-LoxP-STOP-LoxP-TdTomato). Immunostaining of E12.5 placentas from Hoxa13^Cre^; Ai14 animals failed to reveal any RUNX^ + ^c-Kit^ +^ HSPCs that were TdT^ +^ (0 out of 200 total RUNX^ + ^c-Kit^ +^ cells identified in 20 sections derived from 6 placentas) (Fig 3A–C). To address this question with greater sensitivity, we next used flow cytometry to detect CD34^med^c-Kit^ +^ HSPCs that express TdT and therefore are of placental origin. Consistent with our histologic analysis, flow cytometry detected only 0.25% CD34^med^c-Kit^ +^ cells that were TdT^ +^ in the E12.5 Hoxa13^Cre^; Ai14 placenta (Figs 3D, E and S7A).

Since the fetal liver serves as a niche for HSPC expansion during mid-gestation and HSPC numbers in the placenta fall after E12.5, we reasoned that HSPCs of placental origin might exit the placenta and found primarily in the fetal liver after that time point. To address this possibility, we examined c-Kit^ +^ HSCs cells in the E14.5 fetal liver of Hoxa13^Cre^; Ai14 and control Ai14 embryos. In contrast to Cdh5-Cre lineage tracing studies performed previously [43], immunostaining revealed no TdT^ + ^c-Kit^ +^ HSCs cells in the fetal liver (Fig 3F). We next used flow cytometry to examine the contribution of the placenta to the highly characterized HSPC pool in the fetal liver. Analysis of phenotypic long-term repopulating HSCs (Lin^−^c-Kit^+^Sca1^ + ^CD48^−^CD150^+^) in E14.5 Hoxa13^Cre^; Ai14 fetal liver revealed none that were TdT^ +^ (Figs 3G, H and S7B). These studies support the conclusion that placental ECs do not give rise to HSPCs that can be detected in situ in the placenta or subsequently in the fetal liver.

### Colony forming assays reveal Hoxa13^Cre^ marked HSPCs but not placental HECs

Potential limitations of the experiments described above are the limited sensitivity of assays, including immunostaining of placental tissue samples or flow cytometry, to detect potentially rare HSPCs that arise from Hoxa13^ +^ precursors. Therefore, as an orthogonal approach we isolated the TdT^ +^ and TdT^−^ populations of c-Kit^ +^ fetal liver cells and performed colony assays. Approximately 2% of E14.5 fetal liver c-Kit^ +^ cells were TdT^ + ^ and these cells successfully gave rise to different types of hematopoietic cell colonies similar to those generated by c-Kit^ + ^; TdT^−^ cells (Fig 3I, J). Thus, sensitive colony forming assays could indeed identify a small fraction of fetal liver HSPCs derived from Hoxa13^Cre^ labeled precursors. Finally, we examined postnatal bone marrow for placenta-derived hematopoietic cells. Immunostaining of postnatal day 0 (P0) femoral bone from Hoxa13^Cre^; Ai14 pups revealed no CD45^ +^ hematopoietic cells that were TdT^ +^ (Fig 3K).

**Fig 4:**
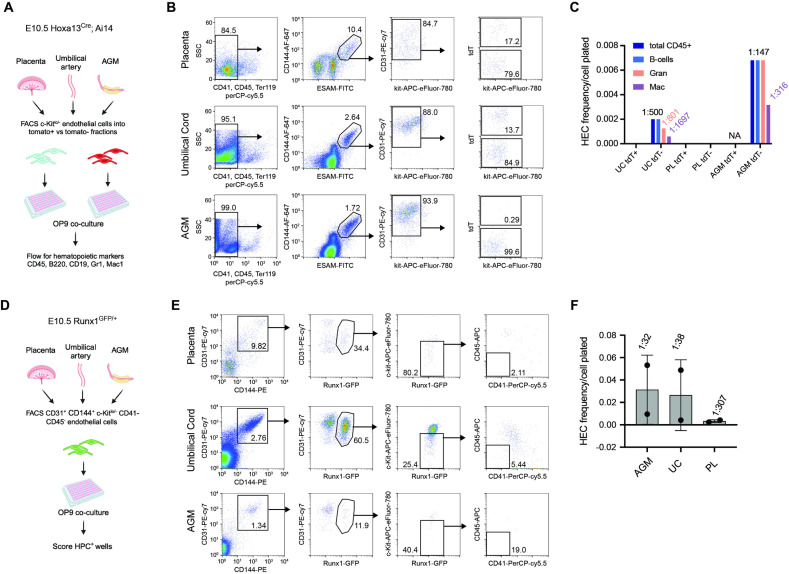
Measurement of placental hemogenic endothelium using ex vivo HEC assays. (**A**) Schematic of the OP-9 co-culture procedure used to analyze HECs capable of differentiating into blood cells ex vivo. (**B**) Placental labyrinth, umbilical cord (UC) and AGM were dissected from Hoxa13Cre; Ai14 E10.5 embryos (*n* = 6) and ECs (CD41^lo/−^CD45^−^Ter119^−^ESAM^+^CD144^ + ^CD31^ + ^c-Kit^lo/−^) fractioned into TdT^ +^ and TdT^−^ fractions. Sorted cells (placenta TdT^ + ^/TdT^−^ =  1,136/2000 cells; UC TdT^ + ^/TdT^−^ =  319/1817 cells; AGM TdT^ + ^/TdT^−^ =  0/2000 cells) were plate in limiting range in five replicates, six dilutions. (**C**) Graph showing frequency of HECs in each sorted population. Frequencies are shown on top of each bar. (**D**) Experimental design. Placental labyrinth, umbilical cord and AGM were dissected from Runx1^IRES-GFP/ +^ E10.5 embryos (*n* = 30). HPC^ +^ refers to wells containing round hematopoietic cells by visual inspection. (**E**) Sorted GFP^ +^ cells expressing endothelial markers (CD31^ + ^CD144^ +^ c-Kit^lo/−^CD41^−^CD45^−^) from each tissue (AGM, 600 cells; UC, 153 cells; Placenta, 885 cells) were plated in OP9 co-cultures in limiting range and screened for hematopoietic outgrowth by the appearance of round hematopoietic cells after 9 days. (**F**) The frequency of HECs in the populations described in panel E. HEC frequencies in C and F were determined using ELDA Limiting Dilution Software. UC, umbilical cord; PL, placenta, AGM, aorta–gonad–mesonephros. The schematic diagrams in panels A and D were generated using Biorender (https://www.biorender.com/academic-license). The data underlying this figure can be found in S1 Data.

The 2% of c-Kit^ +^ hematopoietic cells in the mid-gestational fetal liver that arise from Hoxa13^ +^ lineage precursors may reflect the population of TdT^ +^ HECs observed in the umbilical artery histologically (Fig 3L, M). Alternatively, they could arise from a small number of TdT^ +^ HECs in the placenta that were missed in histologic sections. To distinguish between these two possibilities, we performed limiting dilution HEC assays as previously described [44]. We separated Ter119^−^CD41^lo/−^CD45^−^CD144^ + ^ESAM^+^CD31^ + ^c-Kit^lo/−^ ECs from the placentas, UCs, and AGM regions of E10.5 embryos into TdT^ +^ and TdT^−^ fractions (Fig 4A, B) and plated them in a limiting dilution on OP9 stromal cells in the presence of hematopoietic cytokines to determine the frequency of HECs in each population (Fig 4C). HECs capable of giving rise to B cells and/or myeloid cells were present only in TdT^−^ ECs isolated from the UC and AGM region, but not in TdT^ +^ cells from those tissues, or in either population isolated from the placenta. We also performed hemogenic assays using sorted GFP^ +^ ECs from Runx1^IRES-GFP^ mice [45] and found the frequency of HECs in the GFP^ +^ EC population from the placenta was 20 times lower than in the same population sorted from the AGM and UC (Fig 4D–F). Therefore, the vast majority of HECs are not derived from Hoxa13^ +^ precursors, and there are very few functional HECs in the placenta at this time of development.

Failure to detect TdT^ +^ HECs in any tissue could be explained by the low sensitivity of the HEC assay, an extremely low frequency of TdT^ +^ HECs, or by the absence of HECs in ECs marked by Hoxa13^Cre^. To further address this question, we performed additional immunostaining for RUNX and TdT in the Hoxa13^Cre^; Ai14 UC, a portion of which is derived from *Hoxa13*-expressing mesodermal precursors. Immunostaining identified a subpopulation of RUNX^ +^ cells that appeared to be budding from HECs in the UC that were TdT^ +^ (Fig 3L, M). These findings suggest that a small proportion of HECs in the UC are derived from cells that once expressed Hoxa13, and furthermore that the low level of TdT^ +^ CFUs detected in the E14.5 blood/fetal liver most likely arise from Hoxa13^Cre^-labeled umbilical arterial HECs.

### Timed Cdh5-CreER^T2^ lineage tracing suggests that HSPCs in the placenta arise from hemogenic endothelium in the AGM

**Fig 5:**
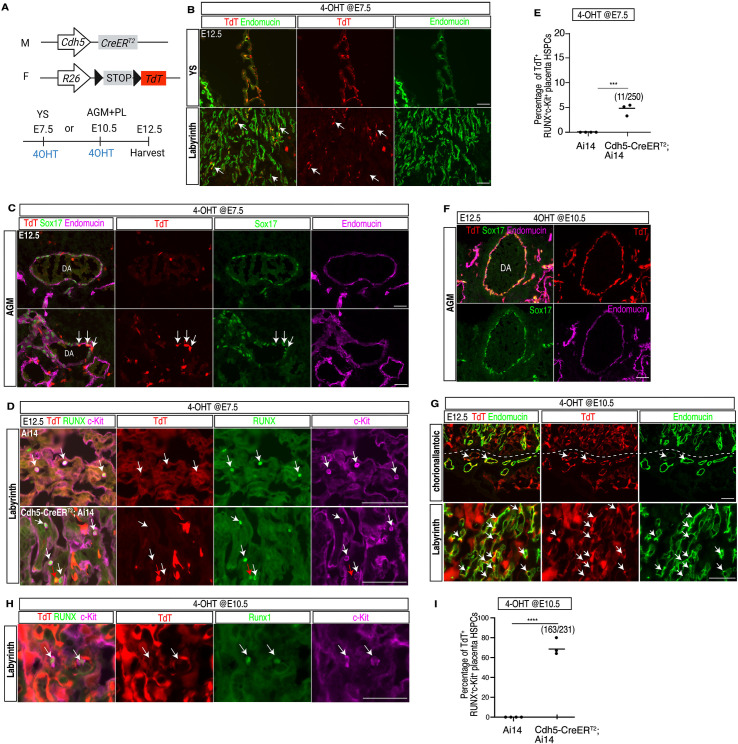
Timed Cdh5-CreER^T2^ lineage tracing of HSPCs in the mouse placenta. (**A**) Tamoxifen administration strategy for timed Cdh5-CreER^T2^ lineage tracing studies. Pregnant dams were orally gavaged with 4-OHT at E7.5 or E10.5 and tissues harvested at E12.5. Anticipated sites of maximal labeling (YS vs. AGM + Placenta) at each time point are indicated. (**B**) Representative images of Endomucin (green) and TdT (red) immunostaining of E12.5 yolk sac (YS, upper) and labyrinth from a Cdh5-CreER^T2^; Ai14 conceptus treated with 4-OHT at E7.5. White arrows indicate rare, TdT^ +^ placental ECs. (**C**) Immunofluorescence staining for TdT (red), Sox17 (green) and Endomucin (magenta) on E12.5 mouse AGM sections following Hoxa13^Cre^ induction at E7.5 (*N* = 3 placentas). DA, dorsal aorta. White arrows indicate rare TdT^ +^ arterial ECs. (**D**) Immunofluorescence staining for TdT (red), RUNX (green) and c-Kit (magenta) on E12.5 mouse placenta sections of Ai14 control (upper panel, *N* = 4 placentas) and Cdh5-CreER^T2^; Ai14 (lower panel, *N* = 3 placentas) following induction with 4-OHT at E7.5. White arrows indicate RUNX^ + ^c-Kit^ +^ cells. Red arrows indicate other RUNX^ + ^ hematopoietic cells. (**E**) Quantification of TdT^ +^ RUNX^ + ^c-Kit^ +^ cells (HSPCs) in the placenta following lineage tracing at E7.5. The absolute numbers of TdT^ +^ placental RUNX^ + ^c-Kit^ +^ cells out of the total number of placental RUNX^ + ^c-Kit^ +^ cells counted are shown. (**F**) Immunofluorescence staining for TdT (red), Sox17 (green) and Endomucin (magenta) on E12.5 mouse AGM sections following induction at E10.5 (*N* = 3 placentas). (**G**) Representative images of Endomucin (green) and TdT (red) immunostaining on E12.5 placenta chorioallantoic region (upper) and labyrinth (lower) from a Cdh5-CreER^T2^ Ai14 conceptus treated with 4-OHT at E10.5. White arrows indicate larger vessels in the chorioallantoic region (top) or placental labyrinth ECs (bottom) that were labeled. The dotted line denotes the border between the labyrinth and chorioallantoic region of the fetal placenta. (**H**) Immunofluorescence staining for TdT (red), RUNX (green) and c-Kit (magenta) on E12.5 mouse placenta sections of a Cdh5-CreER^T2^; Ai14 (*N* = 3 placentas) conceptus treated with 4-OHT at E10.5. White arrows indicate RUNX^ + ^c-Kit^ +^ cells in the placenta. (**I**) Quantification of TdT^ +^ RUNX^ + ^c-Kit^ +^ cells in the placenta following lineage tracing at E10.5. 163/231 indicates the number of TdT^ +^ cells per number of RUNX^ + ^c-Kit^ +^ cells counted. Scale bars: 100 μm (B, D and H), 50 μm (C, F and G). The data underlying this figure can be found in S1 Data.

The studies described above suggested that the RUNX^ + ^c-Kit^ +^ HSPCs identified in the placenta mostly likely arise from the embryo proper and reach the placenta via the circulation. To test this hypothesis, we next performed a series of timed lineage tracing studies that define the progeny of vascular ECs using the well characterized pan-endothelial Cdh5-CreER^T2^ transgene and the Ai14 reporter allele (Fig 5A) [46,47]. Induction of Cdh5-CreER^T2^ activity at E7.5 when yolk sac is hemogenic, followed by tissue analysis at E12.5 revealed TdT expression in most yolk sac ECs (Fig 5B), but only in a very small number of ECs in the AGM region or the placental labyrinth (Figs 5B, C, S8 A–C). Approximately 5% of RUNX^ + ^c-Kit^ +^ cells in the placenta were labeled (11/250, *n* = 3) (Fig 5D, E). E7.5 tamoxifen injection also labeled most placental fetal macrophages, consistent with our recent finding that placental fetal macrophages derive from the yolk sac in a manner similar to other tissue resident macrophages [26] (S8B Fig). We also found that E7.5 Cdh5-CreER^T2^ induction labeled most parietal trophoblast giant cells that border the maternal and fetal parts of the placenta as well as some junctional zone trophoblasts that either remained in the junctional zone or migrated into the decidual region (S8C Fig), consistent with the known expression of *Cdh5* in trophoblasts [48].

In contrast, Cdh5-CreER^T2^ lineage tracing performed following tamoxifen administration at E10.5 when the yolk sac vasculature is not hemogenic, labeled the majority of ECs in the AGM and placenta (Fig 5F, G). Consistent with our previous study showing an essential role for CDH5 in trophoblast remodeling of maternal spiral arteries [48], we detected strong TdT expression in invasive trophoblast cells within the decidua that surround maternal blood vessels (S9A, B Fig). We also detected TdT reporter activity specifically in MCT1^ +^ SynT-I trophoblasts and S-TGCs, but not in MCT4^ +^ SynT-II trophoblasts (S9C Fig). E10.5 tamoxifen administration labeled approximately 70% of the RUNX^ + ^c-Kit^ +^ cells observed in the placenta (163/231, n = 3) (Fig 5H, I). These data suggest that HSPCs present in mouse placenta are primarily derived from ECs in the AGM and/or other sites of hemogenic endothelium within the embryo proper.

### Human placental ECs do not express RUNX or other hemogenic genes

**Fig 6:**
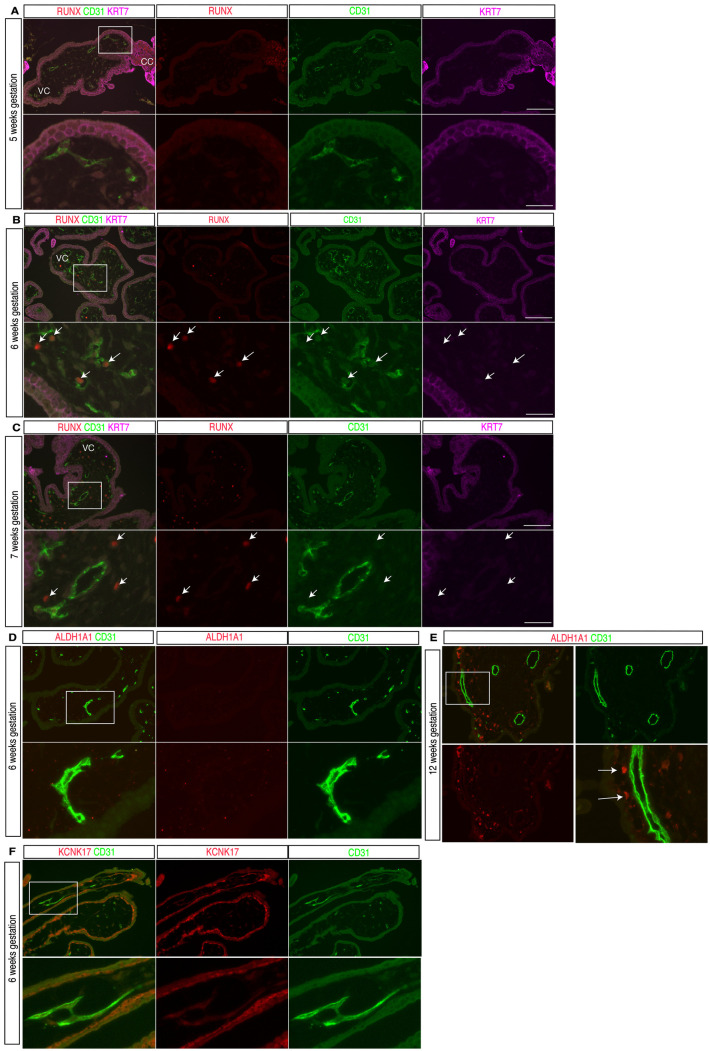
Expression of HEC marker genes in the first-trimester human placenta. (**A**) Immunofluorescence staining for RUNX (red), CD31 (green) and KRT7 (magenta) on human placenta sections of 5 weeks gestational age (3 sections for *N* = 1 placenta). Lower panels show enlarged views of the boxed region in the upper left panel. VC, villous cord; CC, cytotrophoblast cell column. (**B**) Immunofluorescence staining for RUNX (red), CD31 (green) and KRT7 (magenta) on human placenta sections of 6 weeks gestational age (3 sections per placenta, *N* = 2 placentas). Lower panels show enlarged view of the boxed regions in the upper panels. Arrows indicate RUNX^ +^ hematopoietic cells encircled by CD31^ +^ ECs. VC, villous cord. (**C**) Immunofluorescence staining for RUNX (red), CD31 (green) and KRT7 (magenta) on human placenta sections of 7 weeks gestational age (3 sections per placenta for *N* = 2 placentas). Lower panels show enlarged view of the boxed region in the upper panel. Arrows indicate RUNX^ +^ cells in the perivascular, stromal region of the villi. VC, villous cord. (**D**) Immunofluorescence staining for ALDH1A1 (red) and CD31 (green) on human placenta sections of 6 weeks gestational age (*N* = 2 placentas). Lower panels show enlarged view of the boxed region in the upper panel. (**E**) Immunofluorescence staining for ALDH1A1 (red), and CD31 (green) on human placenta sections of 12 weeks gestational age (3 sections per placenta for *N* = 2 placentas). Lower panels show enlarged view of the boxed regions in the upper panels. Arrow indicates abluminal ALDH1A1^ +^ Hofbauer cells within the villi. (**F**) Immunofluorescence staining for KCNK17 (red), and CD31 (green) on human placenta sections of 6 weeks gestational age (3 sections per placenta for *N* = 2 placentas). Lower panels show enlarged view of the boxed regions in the upper panels. Scale bars, 100 μm for upper panels in A, B, C, D and upper three images in E. Twenty μm for lower panels in A, B, C, D and lower three images in E.

To determine whether our findings in the mouse placenta accurately reflect those in the human placenta, we next examined expression of requisite hemogenic endothelial transcription factor RUNX1. Previous studies showed that HSPCs are detected in the human placenta as early as 6 weeks of gestational age and remain present after that time point during gestation [8]. We therefore performed immunostaining for RUNX proteins to detect HECs, KRT7 for trophoblasts and CD31 for all ECs on sections of villi from human placentas of 5, 6, 7 and 12 weeks of gestational age (spanning the first trimester). At 5 weeks gestational age, a time point prior to the appearance of HSPCs in human placenta, no RUNX staining was detected within the placenta villi (0 out of 300 villous ECs in three sections) (Fig 6A). At 6 weeks gestational age, RUNX was detected only in hematopoietic cells within the lumen of fetal blood vessels of the villi, while the placental ECs were RUNX negative (0 out of 250 villous ECs in three sections) (Fig 6B). At 7 and 12 weeks, there remained no RUNX staining in placental ECs (0 out of 300 villous ECs in three sections for each stage), and the majority of RUNX-expressing cells were in mesenchymal region of the placenta villi (Figs 6C and S10A). Further co-staining revealed that these RUNX positive cells were CD68^ +^ Hofbauer cells (known as placenta fetal macrophages in mice) (S10B Fig). To further examine RUNX1 expression in early human placenta villi, we analyzed publicly available scRNA-seq data from first-trimester (6–12 weeks) human placenta villi [49] (S10C Fig). Consistent with the immunostaining findings above, RUNX1 expression was detected in CD68^ +^ hematopoietic cells but not in placenta villous ECs (S10H, I Fig). We also performed immunostaining of human placental tissue at 6–7 weeks of gestation for two other human hemogenic EC markers, ALDH1A1 and KCNK17 [50]. ALDH1A1 was detected in Hofbauer cells as previously reported [51], but not in ECs within the placenta villi (Fig 6D, E). KCNK17 expression was detected in cytotrophoblast cells, but not in placental ECs (Fig 6F). These findings demonstrate that the established HEC gene signature (RUNX1/KCNK17/ALDH1A1) observed in the AGM is not seen in first-trimester human placenta endothelium. These human placental data are consistent with those from the mouse placenta and support the conclusion that neither the human nor the mouse placental endothelium is hemogenic.

## Discussion

Clearly defining the sites of HSPC generation in the developing embryo is necessary to understand the mechanisms that guide that process and how they may be harnessed for therapeutic purposes. HSPCs are found in abundance in both the mouse and human placenta [8,9], and the placenta endothelium has been proposed to be an important hemogenic tissue that contributes to the final HSPC pool [52]. Our studies combine the use of new genetic tools with in vivo and ex vivo approaches to identify the cells that emerge from the placental vasculature and test this hypothesis. Mouse genetic lineage tracing studies demonstrate that placental HSPCs arise from sites of hemogenic endothelium within the embryo proper, but not from placental endothelium. These mouse data are highly consistent with the lack of RUNX^+^ALDH1A1^ + ^KCNK17^ +^ HECs in the human placenta and support a model in which the placenta serves as a site where HSPCs that emerge from the embryo proper expand prior to migrating to the fetal liver and participating in definitive hematopoiesis.

The strongest evidence supporting the conclusion that the placenta is not a site of HSPC emergence from HECs is the use of in vivo, non-invasive genetic lineage tracing using the Hoxa13^Cre^ allele that does not rely upon detection of a molecular marker or ex vivo analysis after manipulation of the embryo and placenta [19]. Immunostaining for the Cre reporter TdT identified very few Hoxa13^Cre^ lineage-traced HSPCs in the placenta, fetal liver or bone marrow, findings consistent with our failure to detect RUNX^ +^ HECs in either the mouse placenta (Fig 2A, B) or the first-trimester human placenta (Fig 6). Since the sample of size of HSPCs examined using immunostained tissue sections is limited, we performed ex vivo colony forming assays to detect Hoxa13^Cre^ lineage HSPCs with maximal sensitivity. These studies indeed demonstrated that 2% of HSPCs in the E14.5 fetal liver are derived from Hoxa13-expressing progenitors, suggesting either that there exists a small population of placental HECs or that those cells arise from a distinct hemogenic endothelial population that is allantois-derived. Our ability to readily detect umbilical artery HECs that are lineage traced by Hoxa13^Cre^, but not placental HECs in an ex vivo hemogenic endothelium assay support the conclusion that these lineage-traced HSPCs most likely arise from allantois-derived ECs that contribute to the umbilical vessels rather than a histologically occult placental source.

The most compelling prior evidence for primary hemogenic activity within the placenta is from analysis of Ncx1-deficient embryos that lack normal cardiac contractile function, and in which HSPCs could presumably not enter the placenta from other sites in the embryo [17]. Analysis of Ncx1-deficient embryos is limited to E10 and earlier time points due to embryonic death, but Rhodes and colleagues [17] reported the presence of CD41^ +^ cells in placental vessels and were able to culture both erythroid and myeloid colonies from the placentas of wild-type and Ncx1-deficient embryos. There are two ways to reconcile these prior findings with those in the present study. First, it is possible that Ncx1 deficiency does not fully prevent blood flow in E10 embryos, thereby enabling HSPCs to still arrive there from extra-placental sources. Studies of Ncx1-deficient myocardial cells report poorly organized fibrillatory activity [15,16] that in the early embryo may still be sufficient to move some blood into the placenta. Alternatively, the CD41^ +^ cells identified in the colonies grown ex vivo may reflect contamination of the placenta sample with cells from the umbilical cord (UC) or yolk sac during the process of dissection, e.g., due to release of blood containing HSPCs. The combined use of hemogenic endothelial assays and genetic lineage tracing in the present study addresses these potential weaknesses.

Our findings correlate well with the fact that placental HSPCs are observed considerably later than the time point at which HSPCs arise from the AGM during human gestation [21], and suggest that the placenta serves as a site of expansion for HSPCs that have originated elsewhere [53]. They also explain how many mammals achieve definitive hematopoiesis without a placenta. Thus, the present study reconciles mouse and human data and supports a model in which the placenta serves as a site of transient HSPC expansion before they home to the fetal liver to further expand and differentiate. Precisely how the placenta fosters expansion of HSPCs from the embryo, and whether HSPCs that expand in these two niches are qualitatively different or have distinct developmental fates, remains to be determined.

### Limitations of the study

Although the Hoxa13^Cre^ allele used in this study labels virtually all placental ECs, we can never fully rule out the possibility of extremely rare Hoxa13-negative placental ECs that might contribute to the HSPC pool. In addition, our study only addresses HSPC ontogeny in the context of normal mouse/human gestation. It does not exclude the possibility that under other non-physiologic conditions, such as stress, the mammalian placenta vasculature can become hemogenic.

## Methods and procedures

### Mice

B6.Cg-Gt(ROSA)26Sortm14(CAG-tdTomato)Hze/J (Ai14, JAX: 007914) and STOCK Gt(ROSA)26Sortm1(DTA)Jpmb/J (R26-LSL-DTA, JAX: 006331) mice were obtained from Jackson Laboratory. Hoxa13^Cre^, Runx1^IRES-GFP^ (gift of Dr. James Downing) and Cdh5-CreER^T2^ alleles have been previously described [26,45,46]. E0.5 corresponds to the time a vaginal plug was discovered. The pregnant dams were sacrificed at the corresponding embryonic day and the embryo and placenta extracted. All experiments were performed at least twice using different litters and used littermate controls on a mixed background unless otherwise indicated. All animal experiments described followed the Guide for the Care and Use of Laboratory Animals of the National Institutes of Health and were performed in accordance with the approval of the University of Pennsylvania Institutional Animal Care and Use Committee (IACUC No. 806811).

### Human tissue collection

First-trimester human placental villi tissues (5–12 weeks gestational age) were collected from the Penn Family Planning and Pregnancy Loss Center under an IRB that was approved by the University of Pennsylvania (#827072) and informed consent was taken prior to tissue collection. Only intrauterine pregnancies and dates by last menstrual period confirmed by ultrasound were collected. Patients with pre-existing medical conditions were excluded from the study. All experiments were conducted according to the principles of the Declaration of Helskinki.

### Tamoxifen administration

Five milligram 4-OHT (4-Hydroxytamoxifen, H7904, Sigma), was dissolved into 250 µL ethanol and incubated in a shaker at 37 °C for 15 min. The clear solution was further diluted into corn oil and 1.2 mg of 200 µ L above suspension was orally gavaged into E7.5 or E10.5 pregnant dams.

### Histology, immunostaining and RNAScope in situ hybridization (ISH) assay on thin sections

Mouse tissue histology was performed as previously described [26]. Yolk sac, embryo and placentae samples were fixed in 2% or 4% paraformaldehyde in PBS overnight at 4 °C with gentle shaking and embedded in OCT (SAKURA, 4538) after several washes with PBS. Sections of 7–10 or 20 µm thickness were collected and stored at − 80 °C. Cryosections were recovered to room temperature and rehydrated in PBS for immunostaining. The following primary antibodies diluted in IHC-Tek Antibody Diluent pH 7.4 (IHC world, IW-100) were used for immunostaining: rabbit anti-RFP (1:200, Rockland, 600-401-379), rabbit anti-RUNX (1:100, Abcam, ab92336), goat anti-c-Kit (1:200, Abcam, AF1356), goat anti-Endomucin (1:200, R&D, AF4666), rat anti-Endomucin (1:100, Abcam, ab106100), goat anti-CD45 (1:200, R&D, AF114), rat anti-F4/80 (1:100, Abcam, ab6640), chicken anti-MCT1 (1:200, Sigma, AB1286-I), rabbit anti-MCT4 (1:200, Sigma, AB3316P), rat anti-CD34 (1:100, Abcam, ab8158), Rat anti-CD43 (1:100, BD biosciences, 552366), goat anti-Coll-I (1:100, SouthernBiotech, 1310-01) and rat anti-Ki67 (1:100, Thermo Fisher, 14-5698-82). For quantifications, each immunostaining was done in three placentas (*N* = 3) and quantification of the staining was performed on counting 4–5 sections with 80–100 μm interval per placenta. Fresh first-trimester human placental villi tissues were formalin fixed at 4 °C overnight. Tissues were then placed in 70% ethanol and embedded in paraffin before blocks were thinly cut and placed on slides with the aid of the University of Pennsylvania Molecular Pathology and Imaging Core. Paraffin sections were deparaffined and rehydrated in distilled water followed by antigen retrieval using IHC-Tek Epitope Retrieval Solution (IHC world, IW-1100) in a steamer (IHC world, IW-1102). The following primary antibodies diluted in IHC-Tek Antibody Diluent pH 7.4 were used for immunostaining: Mouse anti-CD31 (1:100, AngioBio, 11-016), rabbit anti-CD31 (1:1000, Proteintech, 11265-1-AP), rabbit anti-KCNK17 (NBP1-92041), mouse anti-ALDH1A1 (1:100, R&D, MAB5869-SP), rabbit anti-Runx1+Runx2+Runx3 (anti-RUNX) (1:100, Abcam, ab92336), rat anti-KRT7 (1:200, Abcam, AF1356), mouse anti-CD68 (1:100, R&D, MAB20401). Control sections were always placed on the same slide, treated and stained under identical conditions. For RNAScope assay, RNAScope multiplex fluorescent reagent kit v2 (323100, Advanced Cell Diagnostics) was used for in situ hybridization according to the manufacturer’s instructions. *Gfi1* probe (529611) was used, and immunostaining was done following RNAScope. Images were acquired with X4, X10, or X20 objective on a Nikon 80i Eclipse microscope at the same exposure times using NIS Elements Digital Imaging software. ImageJ (NIH) was used to process all the images after data acquisition.

### Immunostaining of thick sections

One hundred fifty micrometer thick sections were obtained using the Leica vibratome (VT1000 S) and stained in 24 well plate as shown previously [54]. After staining using the primary and secondary antibodies listed above, the sections were mounted on slides with spacers and cleared using an alkaline solution [55]. The images were taken on Leica Stellaris 5 Confocal Microscope at the Department of Cell and Developmental Biology Microscopy Core, University of Pennsylvania and analyzed by Imaris 10.0 (Bitplane).

### Flow cytometry

Single cell suspension of mouse placenta was prepared as previously shown with minor modifications [56]. The dissociated cells were labeled by CD34-FITC and AF700-c-Kit antibodies. E14.5 fetal livers were gently crushed on a 70 μm filter with the rubber end of a 1 mL syringe while passing ice cold PBS over the tissue. Lineage depletion was performed by labeling cells with biotinylated lineage antibodies (B220, CD3e, Gr1, and Ter119, eBioscience) and conjugating with streptavidin-magnetic beads (Miltenyi Biotec), then running individual samples through MACS MS separation columns (Miltenyi Biotec). Flow-through cells were labeled with the following antibodies: B220-, CD3e- Gr1- and Ter119-in eflour-450, kit-APC-ef780, Sca1-Percp-cy5.5, CD48-APC, CD150-PE-cy7 and DAPI (Invitrogen) was used for viability. Samples were analyzed on a BD LSR-II flow cytometer and analyzed by FlowJo software.

### Hemogenic EC (HEC) assays

Placenta labyrinth, UC, and AGM tissues were individually harvested and pooled from E10.5 embryos derived from Hoxa13^Cre^; Ai14 crosses. Cells were labeled with the following antibodies: CD41 PerCp-e710, CD45 PerCp-cy5.5, ESAM-FITC, kit-APC eFluor 780 and Ter119 PerCp-cy5.5, and stained with DAPI. HECs (CD45^−^Ter119^−^CD41^lo/−^VE-Cadherin^ +^ ESAM^+^CD31^ +^ and c-Kit^lo/−^) sub-gated on TdTomato were sorted on a BD Influx. Sorted cells were co-cultured in limiting dilution on OP9 stromal cells in media (alphaMEM, Invitrogen), 10% FBS, Penicillin/Streptomycin and supplemented with 10 ng/mL each mIL-3, mIL-7, mFlt3L and mSCF (Peprotech) for 9 days. Hematopoietic cells labeled with CD45-PE, CD11b-Mac1, Gr1-Percp710 or CD19-APC and or B220-PEcy7 were analyzed on a CytoFlex LX (Beckman Coulter). The antibodies for flow cytometry were from Invitrogen, eBiosciences and BD Pharmingen. Potential GFP + HECs were enriched and sorted from Runx1^IRES-GFP^ mice, as previously shown [57]. Sorted cells were then plated in limiting dilutions with OP9 cells for 9 days in medium containing 10 ng/mL of IL-3, IL-7, Flt3, and SCF. The frequency of HECs, determined by the presence of B, myeloid, or B + myeloid cells after culture by FACS using hematopoietic markers (B220, CD19, Mac1, Gr1, and CD45) was determined using ELDA Limiting Dilution Software [58].

### Analysis of single cell transcriptome data

A single nuclei RNA-seq dataset [33] from wild-type mouse placenta labyrinth was downloaded and analyzed in Seurat v4.0 [59]. We applied SCTransform and clustered following the standard workflow, with feature-level analysis and result visualization scripted with ggplot2, Seurat, and/or ggpubr. A single cell RNA-seq dataset [49] from first-trimester human placenta villi was obtained in R data format (kindly provided by Drs. Suryawanshi and Tuschl, Rockefeller University). We transferred the complete count matrix to a new Seurat object, applied SCTransform-based integration, and followed a similar workflow as above.

### Statistics

All data were analyzed with GraphPad Prism (version 8) and represented as mean ±  SEM. *P*-values were calculated using an unpaired two-tailed Student *t* test or one-way ANOVA plus Tukey post hoc analysis as indicated. *P* values < 0.05 were considered statistically significant and were indicated as follows: * *P* <  0.05; **P <  0.01; ***P <  0.001; and ****P <  0.0001. In S6 Fig, *p*-values were calculated using a one-sample *T* and Wilcoxon test to test the differences between observed expression values and 0. *P*-values in the scRNA-seq analysis in S4 and S10 Figs were shown based on unpaired, two-tailed Welch *t* test with unequal variances.

## Supporting information

S1 Fig c-Kit expression and placenta HSPC characterization. (**A**) Immunostaining for c-Kit (cerulean), MCT1 (red) and MCT4 (green) on E12.5 mouse placenta labyrinth. The lower images show the boxed region in the upper image. White arrows indicate c-Kit^+^MCT1^ +^ S-TGCs and white arrowheads indicate rounded c-Kit^ +^ HSPCs. Scale bars: 50 μm (**B**) Schematic showing structure of E12.5 placenta labyrinth and marker gene expression in different cell types.
(TIF)

S2 Fig Placental HSPC expression of the pre-HSC and multi-potent progenitor marker CD43. (**A**, **B**) Immunostaining of CD43 (magenta), c-Kit (green) and RUNX (red) on E11.5 (**A**) and E12.5 (**B**) mouse placenta sections. Boxed region in upper panel is shown in lower panel. White arrows indicate CD43^ + ^c-Kit^+^RUNX^ +^ HSPCs. Yellow arrows indicate CD43^−^c-Kit^+^RUNX^ +^ HSPCs. (**C**) Quantification of percentage of CD43^ +^ HSPCs in A and B. The number above the bar shows the CD43^ +^ cells/ total HSPCs counted. Scale bars: 50 μm.
(TIF)

S3 Fig Characterization of RUNX expression on thick section of E11.5 placenta. (**A**) 3D Snapshot view showing the labyrinth and CA region of E11.5 mouse placenta thick sections stained for RUNX (red), CD34 (green) and c-Kit (magenta) (3 sections per placenta for *N* = 2 placentas). The RUNX channel is over-exposed to maximize sensitivity as RUNX signal intensity in labyrinth is much weaker compared to its signal intensity in decidual stromal cells. Note the presence of RUNX expression in the CA region does not overlap with CD34^ +^ arterial ECs. The white arrows indicate RUNX^ + ^c-Kit^ +^ hematopoietic clusters in the umbilical artery. The lower panel shows the boxed area in the upper panel. (**B**) 3D Snapshot view showing the decidua and labyrinth region of E11.5 mouse placenta thick sections stained for RUNX (red), CD34 (green), and c-Kit (magenta). Note extensive RUNX staining in decidua stromal cells and sparse RUNX staining in hematopoietic cells within the labyrinth. The lower panel shows the boxed labyrinth region in the upper panel. UC, umbilical cord; Lab, labyrinth, Jz, junctional zone; Dec, decidual.
(TIF)

S4 Fig Runx1–3, Sox17 and Gata2 expression in the mouse placenta. (**A**) UMAP plot of single nuclei RNA-seq analysis of Runx1 expression in combined placenta tissues from different stages (E9.5, E10.5, E12.5 and E14.5) is shown. ECs, decidua stroma cells and hematopoietic cell populations are outlined. (**B**) Runx1 expression levels in decidual and placenta fetal ECs at E9.5, E10.5, E12.5 and E14.5 are shown. (**C**) Dot plot of Runx1, Runx2 and Runx3 gene expression level and percentage in hematopoietic cells, decidual stroma cells and ECs in combined single nuclei RNA-seq database. (**D**) Immunostaining for Sox17 (red), Endomucin (green) and Gata2 (magenta) on E10.5 mouse placenta sections. Dotted lines indicate the boundary between the labyrinth and CA regions. Middle and bottom panels show the boxed #1 and #2 regions in the upper panel. White arrowheads indicate the Crypt of Duval and white arrows indicate arterial ECs that are Sox17^ + ^. A, artery and V, vein. Scale bars: 100 μm. (**E**, **F**) UMAP plot and expression levels for Sox17 in mouse placenta across E9.5, E10.5, E12.5 and E14.5. EC populations are outlined. (**G**, **H**) UMAP plot and expression levels for Gata2. ECs and trophoblast cell populations are outlined. *P*-values were shown based on unpaired, two-tailed Welch *t* test with unequal variances.
(TIF)

S5 Fig *Gfi1* and *Hey2* expression in the mouse placenta. (**A**) Representative image for *Gfi1* mRNA (detected using RNA-scope in red), Endomucin (detected using immunofluorescence in green) and DAPI (blue) staining of an E10.5 mouse embryo section through the dorsal aorta (3 sections per placenta, *N* = 2 placentas). DA, dorsal aorta. D and V indicate dorsal and ventral side of the DA. (**B**) Representative image of *Gfi1* (RNA-scope in red), CD34 (Immunofluorescence in green) and DAPI (blue) staining of E10.5 mouse placenta section. The white dotted line delineates the border between chorioallantoic region (CA) and labyrinth (Lab). (**C**, **D**) UMAP plot and expression of *Gfi1* (C) and *Hey2* (D) in mouse placenta across E9.5, E10.5, E12.5 and E14.5. EC populations are outlined.
(TIF)

S6 Fig Hoxa13^Cre^ lineage tracing in extra-placental vascular beds and Hoxa13 gene expression during murine HSPC ontogeny. (**A**) Diagram of Hoxa13^Cre^ lineage tracing in the allantois and placenta. Red indicates Hoxa13^Cre^ lineage positive cells and tissue. Dotted lines indicate mosaic labeling by Hoxa13^Cre^ in the umbilical cord. ECs, endothelial cells; Lab, labyrinth; CA, chorioallantoic region; UC, umbilical cord; MCs, mesenchymal cells. (**B**) Immunostaining for TdT (red), CD34 (green) and Endomucin (magenta) in mouse embryo tissues in the E11.5 AGM (upper), brain (middle) and UC (umbilical cord, bottom). D and V in top panel indicate dorsal and ventral side; A and V in lowest panel indicate umbilical artery and umbilical vein. (**C**) Immunostaining for Endomucin (red) and MCT1 (green) in mouse placenta sections from E10.5 R26-LSL-DTA^ + /0^ control (upper panel) and Hoxa13^Cre/ + ^; R26-LSL-DTA^ + /0^ animals (lower panel). Lab, labyrinth. Boxed region in the far left image is shown at higher magnification on the right. (**D**) Expression levels of the Hoxa13 gene in HSPCs sorted from different tissues during mouse HSPC ontogeny extracted from the StemSite portal (http://daleystem.hms.harvard.edu/) [42]. ND, not detected. ESCs, embryonic stem cells; EB, embryoid body; YS, yolk sac; PL, placenta; FL, fetal liver; WBM, whole bone marrow; HSCs, hematopoietic stem cells. (**E**) Expression levels of the positive control gene Hoxa9 in HSPCs during mouse ontogeny obtained using the same source as in (**D**). Each dot indicates one sample. *P*-values were calculated using a one-sample *T* and Wilcoxon test to test the differences between observed expression values and 0 (**p < 0.01; ***p < 0.005, ****p < 0.0001). Scale bars are all 150 μm except for the second row of AGM staining in (**A**) is 50 μm.
(TIF)

S7 Fig FACS plots of placental and fetal liver HSPCs from control animals. (**A**) FACS plot of E12.5 control Ai14 placenta for HSPCs. Red gate and text indicates TdT^ +^ cells. (**B**) FACS plot of HSPCs from E14.5 control Ai14 fetal livers.
(TIF)

S8 Fig Lineage tracing of Cdh5-CreER^T2^; Ai14 cells following induction with 4-OHT at E7.5. (**A**) Whole placenta immunostaining of TdT (red), CK8 (green) and Endomucin (magenta) of E12.5 Cdh5-Cre^ERT2^; Ai14 animals treated with 4-OHT at E7.5. (**B**) Immunostaining for TdT (red), F4/80 (green) and CD45 (magenta) on the placenta labyrinth area of E12.5 Cdh5-CreER^T2^; Ai14 animals. The lower panel shows the boxed region in the upper panel at higher magnification. White arrowheads indicate placenta fetal macrophages. (**C**) Immunostaining of TdT (red), CK8 (green) and Endomucin (green) on E12.5 Cdh5-CreER^T2^; Ai14 animals showing the boundary between decidua and junctional zone. White arrows indicate invasive trophoblast cells. Dotted lines indicate the separation between maternal and fetal sides of the placenta. The lower image shows the boxed region above at higher magnification. Scale bars: 500 μm (A), 100 μm (upper C), 50 μm (B, bottom C).
(TIF)

S9 Fig Lineage tracing of Cdh5-CreER^T2^; Ai14 cells following induction with 4-OHT at E10.5. (**A**) Whole placenta immunostaining for TdT (red), CK8 (green) and Endomucin (magenta) of E12.5 Cdh5-CreER^T2^; Ai14 animals treated with 4-OHT at E10.5. (**B**) Immunostaining of TdT (red), CK8 (green) and Endomucin (magenta) on E12.5 Cdh5-CreER^T2^; Ai14 animals showing the boundary between decidua and junctional zone. White arrows indicate invasive trophoblast cells. Dotted lines indicate the separation between maternal and fetal sides of the placenta. The lower images show the boxed region in the panel above at higher magnification. (**C**) Immunostaining of TdT (red), MCT1 (green) and MCT4 (magenta) on E12.5 Cdh5-CreER^T2^; Ai14 animals showing the labyrinth area in the placenta. The stars indicate S-TGCs. The arrows indicate TdT^ +^ SynT-I cells. (**D**) Immunostaining of TdT (red) and Endomucin (green) on E12.5 Cdh5-CreER^T2^; Ai14 animals showing the yolk sac area. 500 μm (**A**), 100 μm (**C**, **D**), 50 μm (**B**).
(TIF)

S10 Fig RUNX gene expression in first trimester human placenta villi. (**A**) Immunofluorescence staining for RUNX (red), CD31 (green) and KRT7 (magenta) on 12 week gestational age human placenta sections (3 sections for *N* = 1 placenta). Lower panels show enlarged view of the boxed regions in the upper panels. Arrows indicate RUNX^ +^ cells in the perivascular region of the villi. vc, villous cord. (**B**) Immunofluorescence staining for RUNX (red) and CD68 (green) on 12 week gestational age human placenta sections (3 sections for *N* = 1 placenta). Three bottom panels show enlarged view of the boxed region in three upper panels. Arrows indicate RUNX^+^CD68^ +^ Hofbauer cells. (**C**) Plot showing cluster integration of scRNA-seq dataset from first trimester human placenta villi. (**D**) UMAP plot showing CD31/PECAM1 expression in villus ECs. The EC population is outlined. (**E**) Plot showing quantification of CD31 expression in Hofbauer cells and ECs. (**F**) UMAP plot showing CD68 expression in Hofbauer cell populations. Hofbauer cell populations are outlined. (**G**) Plot showing quantification of CD68 expression in Hofbauer cells and ECs. (**H**) UMAP plot showing RUNX1 expression in Hofbauer cells. (**I**) Plot showing no RUNX1 expression in ECs and significant RUNX1 expression in Hofbauer cells. *P*-values were shown based on unpaired, two-tailed Welch *t* test with unequal variances.
(TIF)

S1 Data Raw data files for all the quantifications used in the study. (XLSX)

## Abbreviations

AGM: aorta–gonad–mesonephros
CA: chorioallantoic
DTA: diphtheria toxin fragment A
E: embryonic day
EHT: endothelial-to-hematopoietic transition
HECs: hemogenic endothelial cells
HPs: hematopoietic progenitors
HSPCs: hematopoietic stem and progenitor cells
S-TGCs: sinusoidal-trophoblast giant cells
UC: umbilical cord
