# Supplementary figures and images for: Lineage tracing studies suggest that the placenta is not a de novo source of hematopoietic stem cells
Source: PLoS Biol. 2025 Jan 28;23(1):e3003003. doi: 10.1371/journal.pbio.3003003 (PMC11774391; doi:10.1371/journal.pbio.3003003)

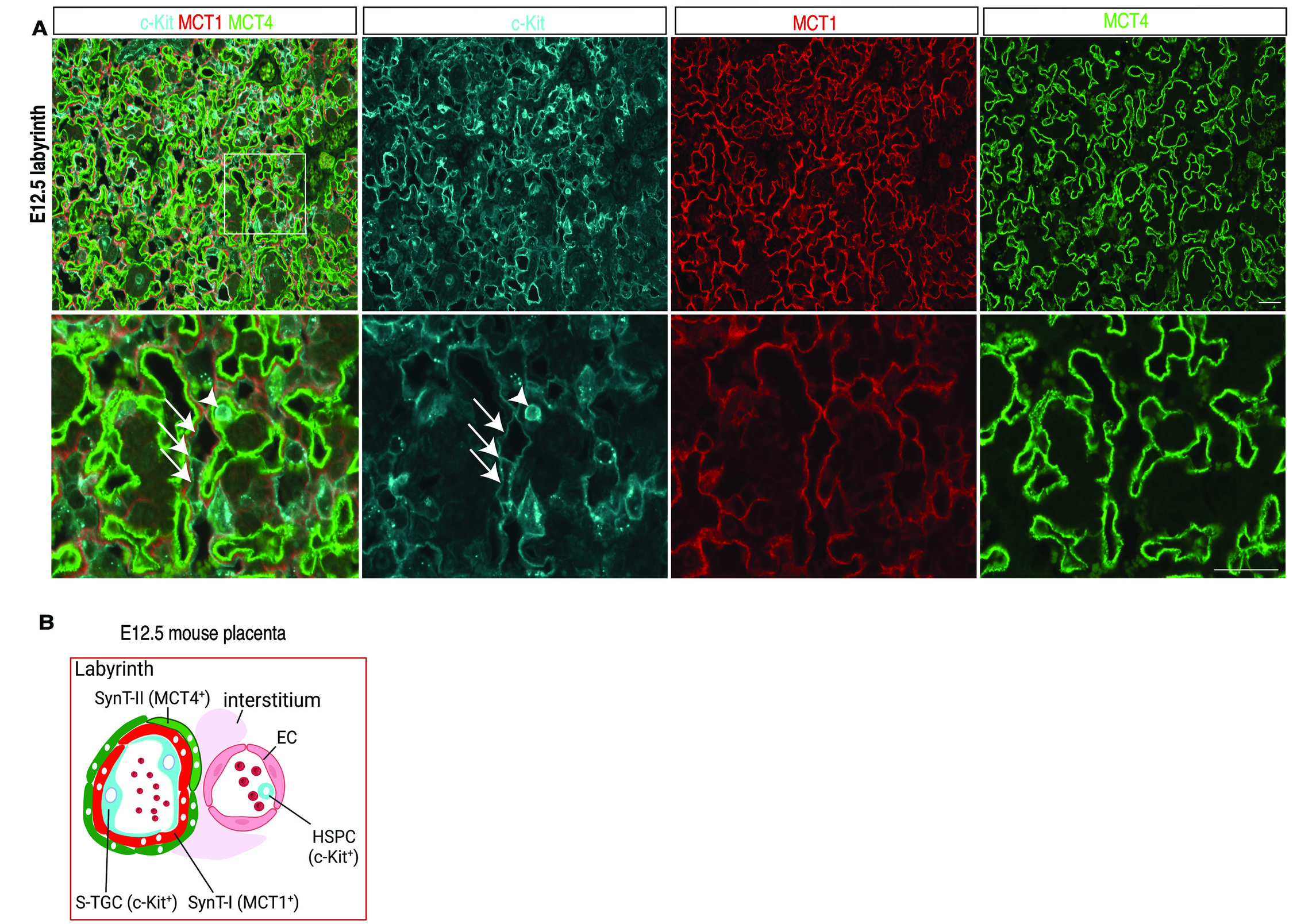

Supplement: S1 Fig — (A) Immunostaining for c-Kit (cerulean), MCT1 (red) and MCT4 (green) on E12.5 mouse placenta labyrinth. The lower images show the boxed region in the upper image. White arrows indicate c-Kit+MCT1 + S-TGCs and white arrowheads indicate rounded c-Kit + HSPCs. Scale bars: 50 μm (B) Schematic showing structure of E12.5 placenta labyrinth and marker gene expression in different cell types. (TIF) [file pbio.3003003.s001.tif]

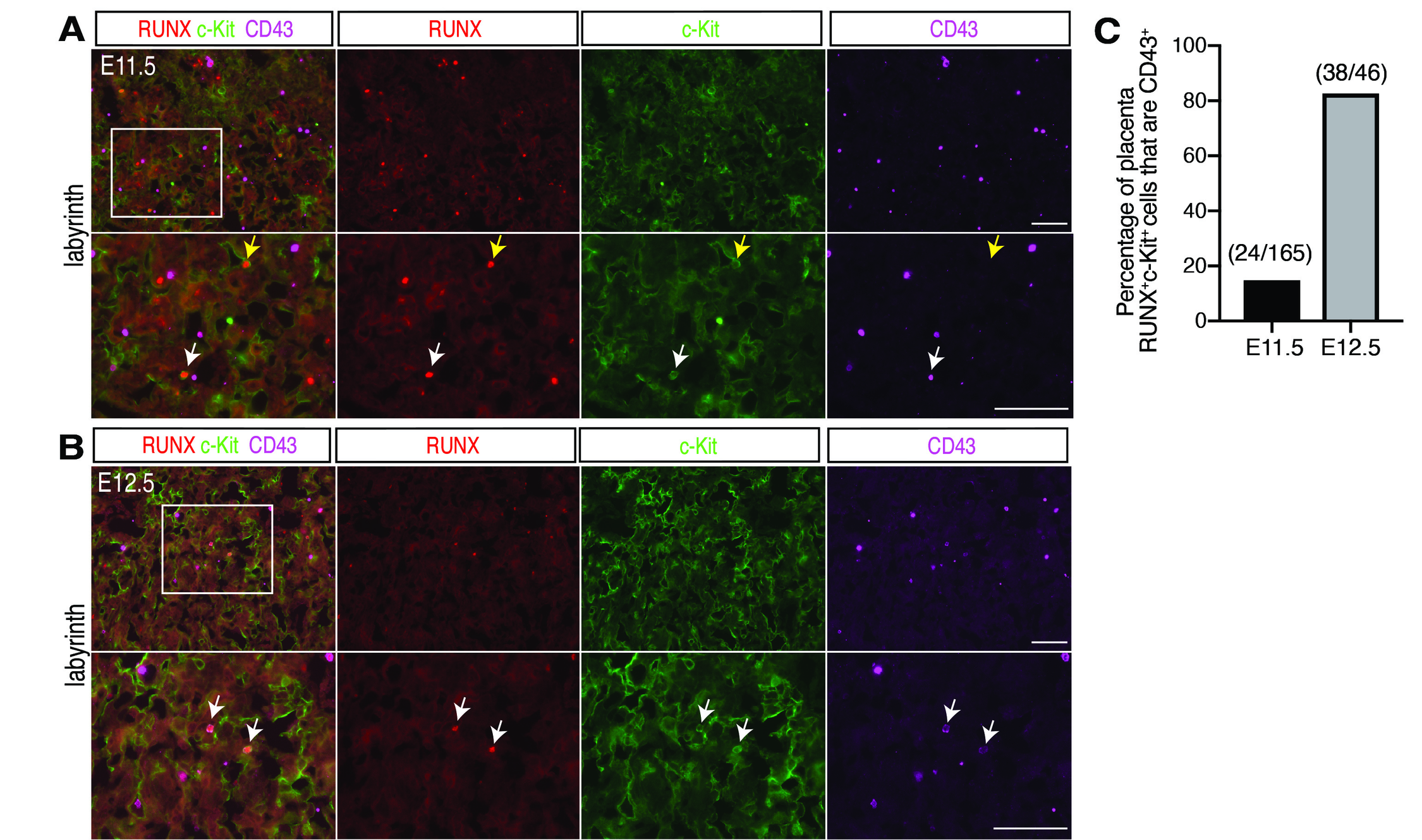

Supplement: S2 Fig — (A, B) Immunostaining of CD43 (magenta), c-Kit (green) and RUNX (red) on E11.5 (A) and E12.5 (B) mouse placenta sections. Boxed region in upper panel is shown in lower panel. White arrows indicate CD43 + c-Kit+RUNX + HSPCs. Yellow arrows indicate CD43−c-Kit+RUNX + HSPCs. (C) Quantification of percentage of CD43 + HSPCs in A and B. The number above the bar shows the CD43 + cells/ total HSPCs counted. Scale bars: 50 μm. (TIF) [file pbio.3003003.s002.tif]

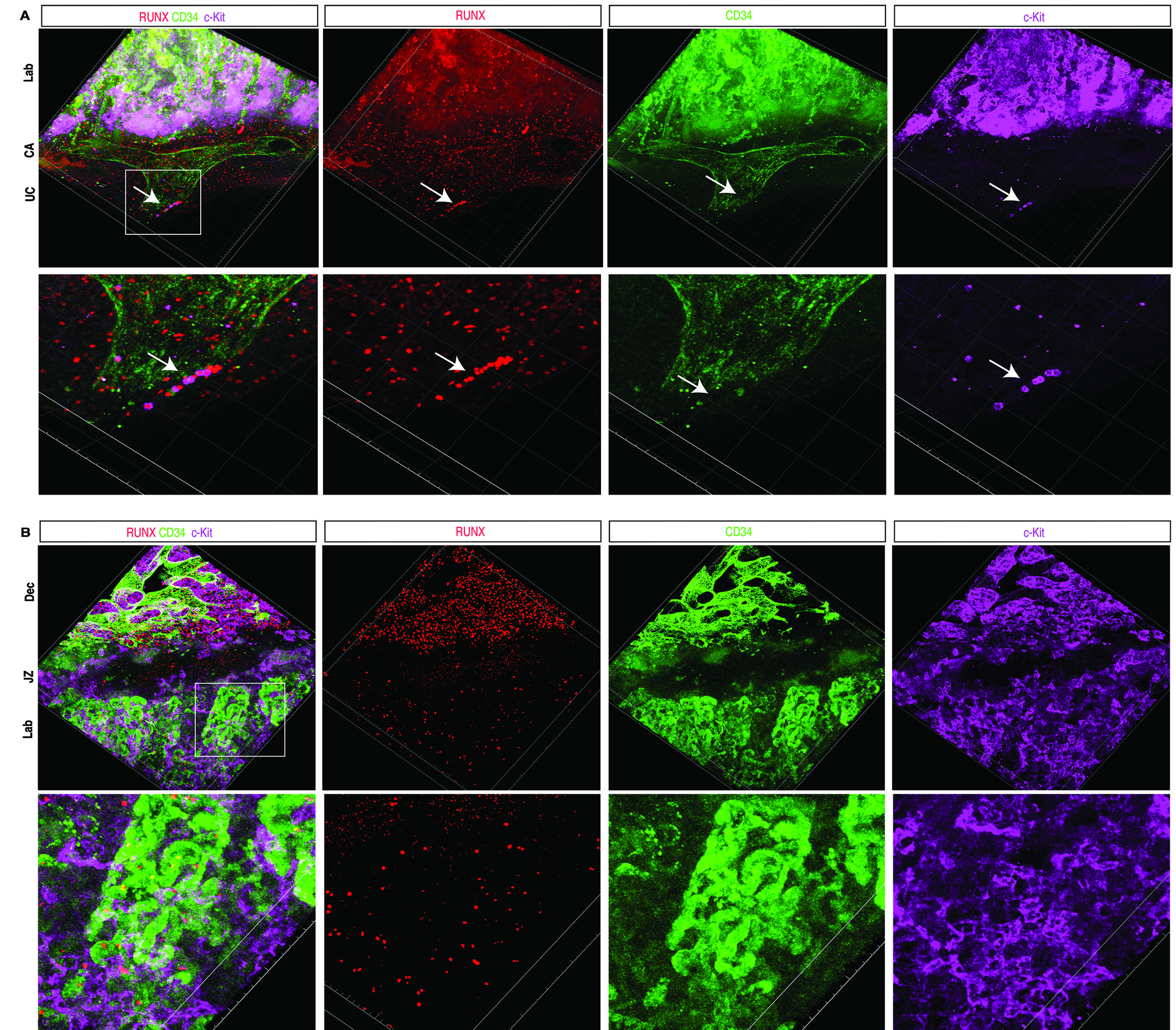

Supplement: S3 Fig — (A) 3D Snapshot view showing the labyrinth and CA region of E11.5 mouse placenta thick sections stained for RUNX (red), CD34 (green) and c-Kit (magenta) (3 sections per placenta for N = 2 placentas). The RUNX channel is over-exposed to maximize sensitivity as RUNX signal intensity in labyrinth is much weaker compared to its signal intensity in decidual stromal cells. Note the presence of RUNX expression in the CA region does not overlap with CD34 + arterial ECs. The white arrows indicate RUNX + c-Kit + hematopoietic clusters in the umbilical artery. The lower panel shows the boxed area in the upper panel. (B) 3D Snapshot view showing the decidua and labyrinth region of E11.5 mouse placenta thick sections stained for RUNX (red), CD34 (green), and c-Kit (magenta). Note extensive RUNX staining in decidua stromal cells and sparse RUNX staining in hematopoietic cells within the labyrinth. The lower panel shows the boxed labyrinth region in the upper panel. UC, umbilical cord; Lab, labyrinth, Jz, junctional zone; Dec, decidual. (TIF) [file pbio.3003003.s003.tif]

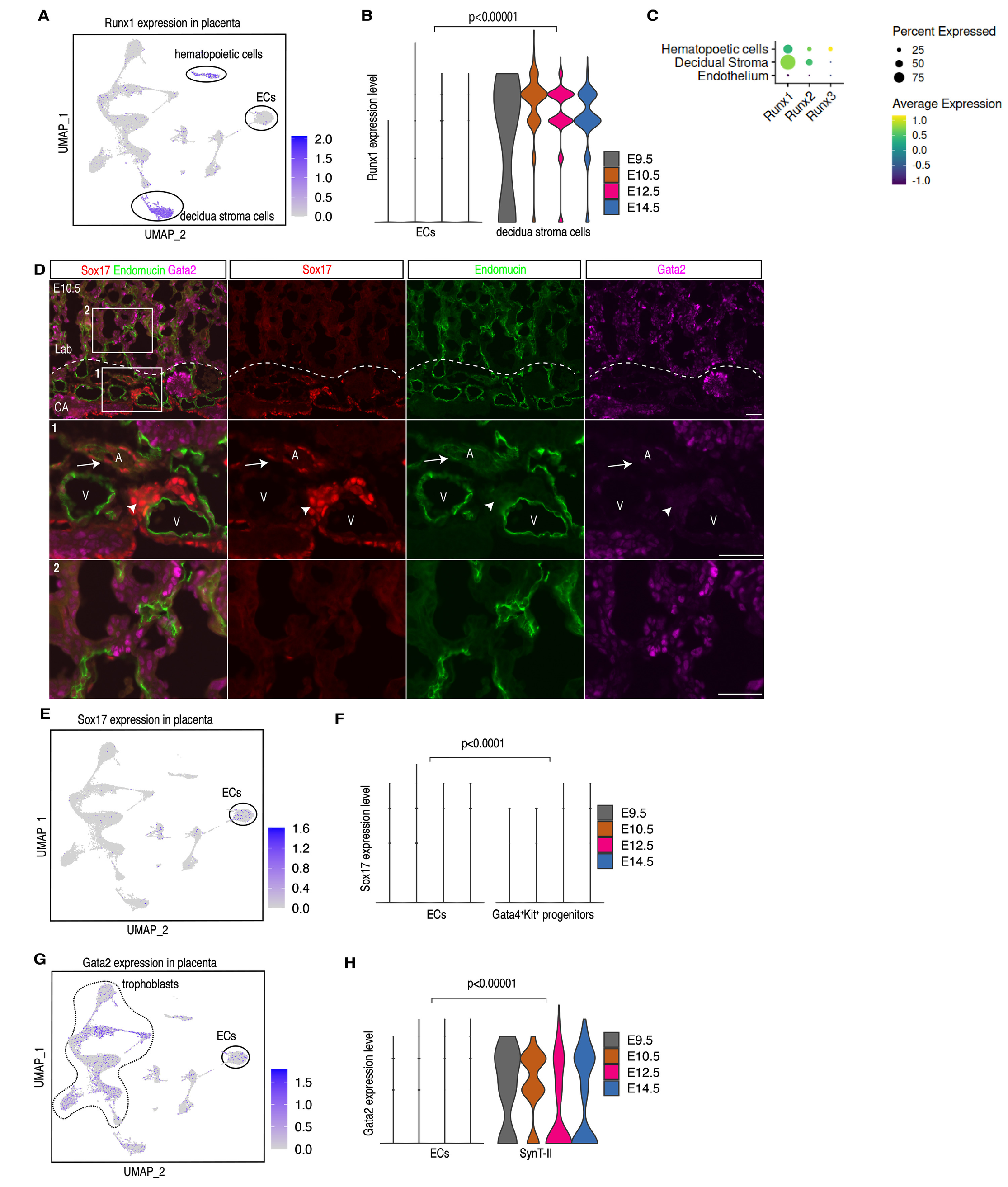

Supplement: S4 Fig — (A) UMAP plot of single nuclei RNA-seq analysis of Runx1 expression in combined placenta tissues from different stages (E9.5, E10.5, E12.5 and E14.5) is shown. ECs, decidua stroma cells and hematopoietic cell populations are outlined. (B) Runx1 expression levels in decidual and placenta fetal ECs at E9.5, E10.5, E12.5 and E14.5 are shown. (C) Dot plot of Runx1, Runx2 and Runx3 gene expression level and percentage in hematopoietic cells, decidual stroma cells and ECs in combined single nuclei RNA-seq database. (D) Immunostaining for Sox17 (red), Endomucin (green) and Gata2 (magenta) on E10.5 mouse placenta sections. Dotted lines indicate the boundary between the labyrinth and CA regions. Middle and bottom panels show the boxed #1 and #2 regions in the upper panel. White arrowheads indicate the Crypt of Duval and white arrows indicate arterial ECs that are Sox17 + . A, artery and V, vein. Scale bars: 100 μm. (E, F) UMAP plot and expression levels for Sox17 in mouse placenta across E9.5, E10.5, E12.5 and E14.5. EC populations are outlined. (G, H) UMAP plot and expression levels for Gata2. ECs and trophoblast cell populations are outlined. P-values were shown based on unpaired, two-tailed Welch t test with unequal variances. (TIF) [file pbio.3003003.s004.tif]

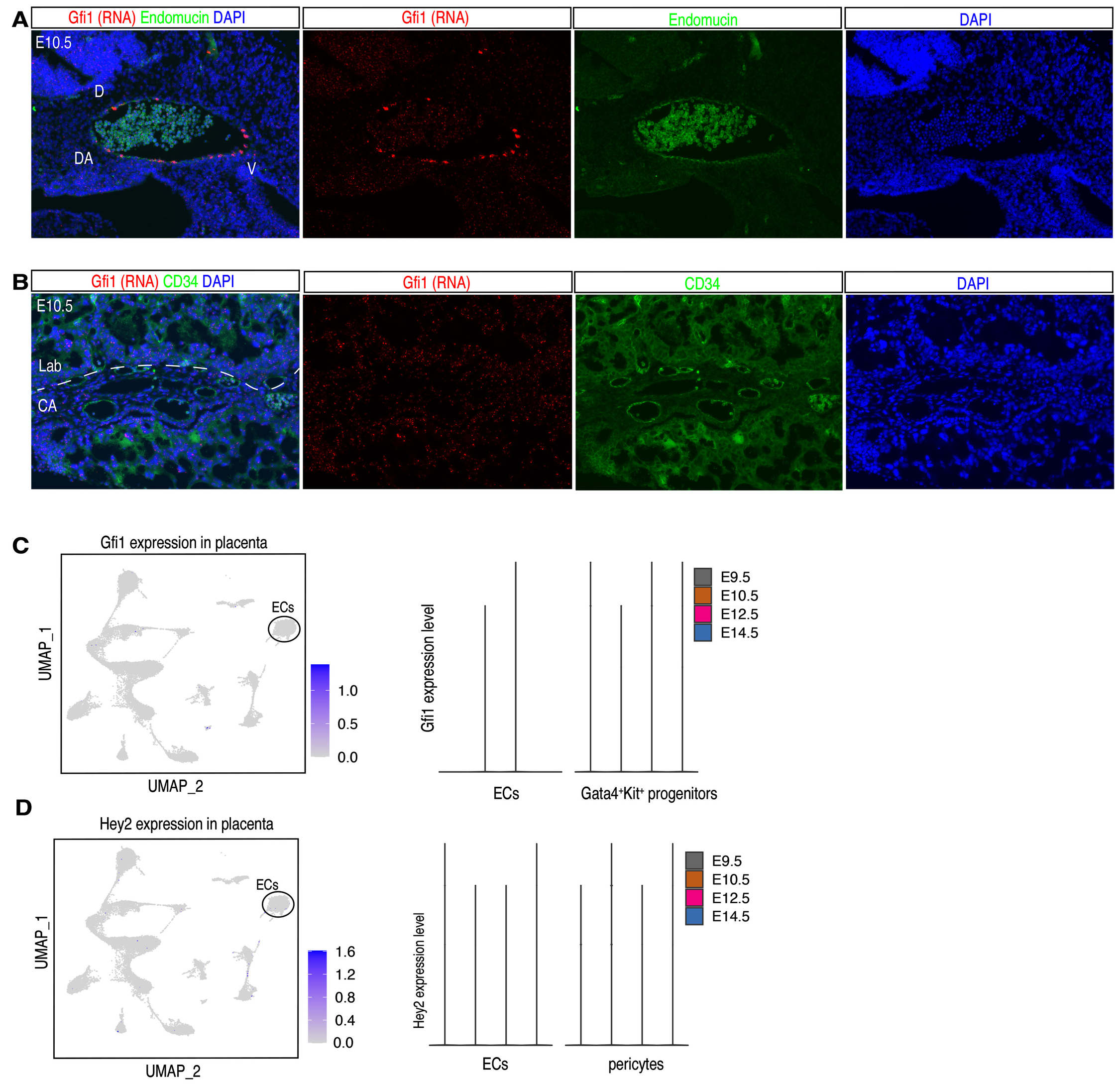

Supplement: S5 Fig — (A) Representative image for Gfi1 mRNA (detected using RNA-scope in red), Endomucin (detected using immunofluorescence in green) and DAPI (blue) staining of an E10.5 mouse embryo section through the dorsal aorta (3 sections per placenta, N = 2 placentas). DA, dorsal aorta. D and V indicate dorsal and ventral side of the DA. (B) Representative image of Gfi1 (RNA-scope in red), CD34 (Immunofluorescence in green) and DAPI (blue) staining of E10.5 mouse placenta section. The white dotted line delineates the border between chorioallantoic region (CA) and labyrinth (Lab). (C, D) UMAP plot and expression of Gfi1 (C) and Hey2 (D) in mouse placenta across E9.5, E10.5, E12.5 and E14.5. EC populations are outlined. (TIF) [file pbio.3003003.s005.tif]

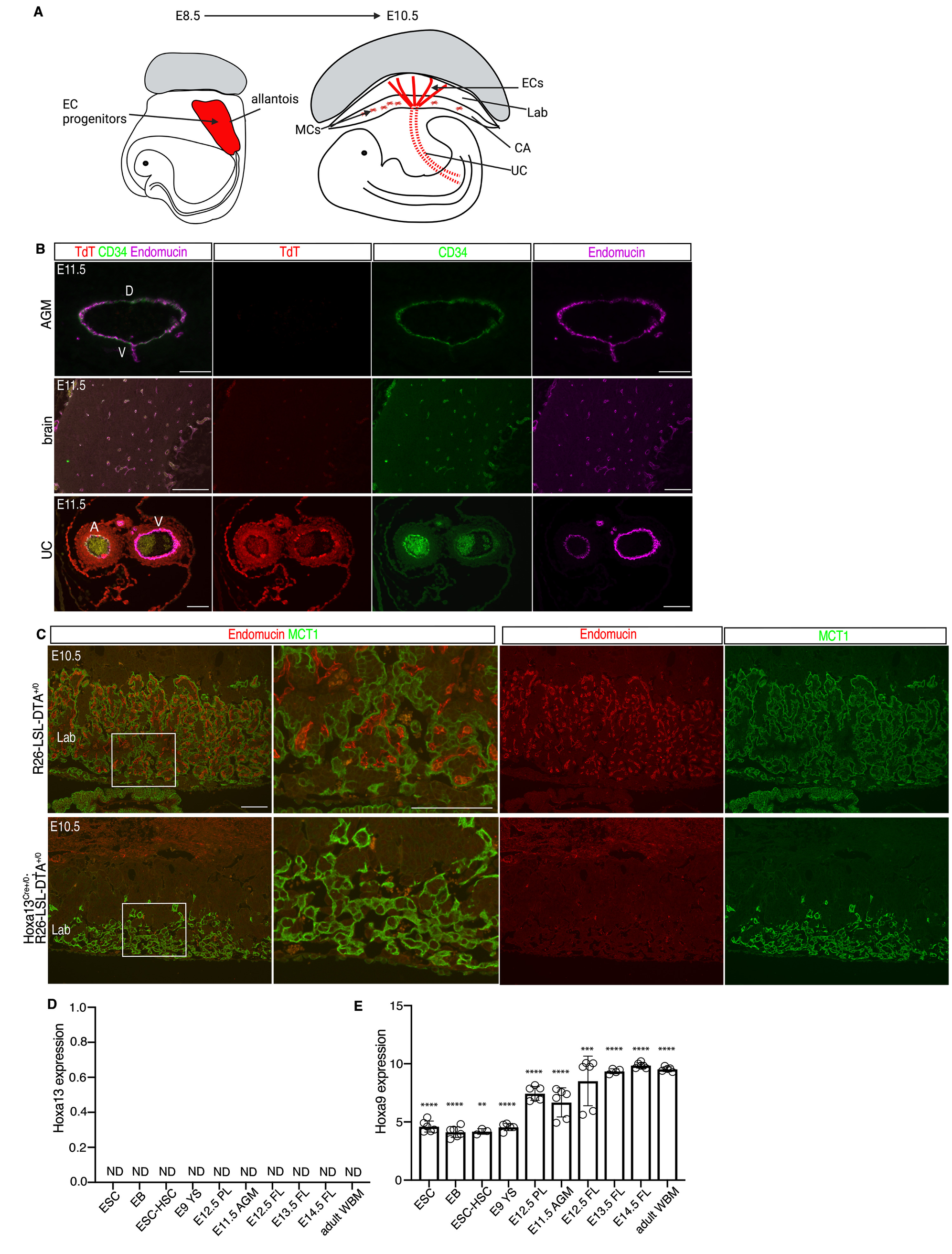

Supplement: S6 Fig — (A) Diagram of Hoxa13Cre lineage tracing in the allantois and placenta. Red indicates Hoxa13Cre lineage positive cells and tissue. Dotted lines indicate mosaic labeling by Hoxa13Cre in the umbilical cord. ECs, endothelial cells; Lab, labyrinth; CA, chorioallantoic region; UC, umbilical cord; MCs, mesenchymal cells. (B) Immunostaining for TdT (red), CD34 (green) and Endomucin (magenta) in mouse embryo tissues in the E11.5 AGM (upper), brain (middle) and UC (umbilical cord, bottom). D and V in top panel indicate dorsal and ventral side; A and V in lowest panel indicate umbilical artery and umbilical vein. (C) Immunostaining for Endomucin (red) and MCT1 (green) in mouse placenta sections from E10.5 R26-LSL-DTA + /0 control (upper panel) and Hoxa13Cre/ + ; R26-LSL-DTA + /0 animals (lower panel). Lab, labyrinth. Boxed region in the far left image is shown at higher magnification on the right. (D) Expression levels of the Hoxa13 gene in HSPCs sorted from different tissues during mouse HSPC ontogeny extracted from the StemSite portal (http://daleystem.hms.harvard.edu/) [42]. ND, not detected. ESCs, embryonic stem cells; EB, embryoid body; YS, yolk sac; PL, placenta; FL, fetal liver; WBM, whole bone marrow; HSCs, hematopoietic stem cells. (E) Expression levels of the positive control gene Hoxa9 in HSPCs during mouse ontogeny obtained using the same source as in (D). Each dot indicates one sample. P-values were calculated using a one-sample T and Wilcoxon test to test the differences between observed expression values and 0 (**p < 0.01; ***p < 0.005, ****p < 0.0001). Scale bars are all 150 μm except for the second row of AGM staining in (A) is 50 μm. (TIF) [file pbio.3003003.s006.tif]

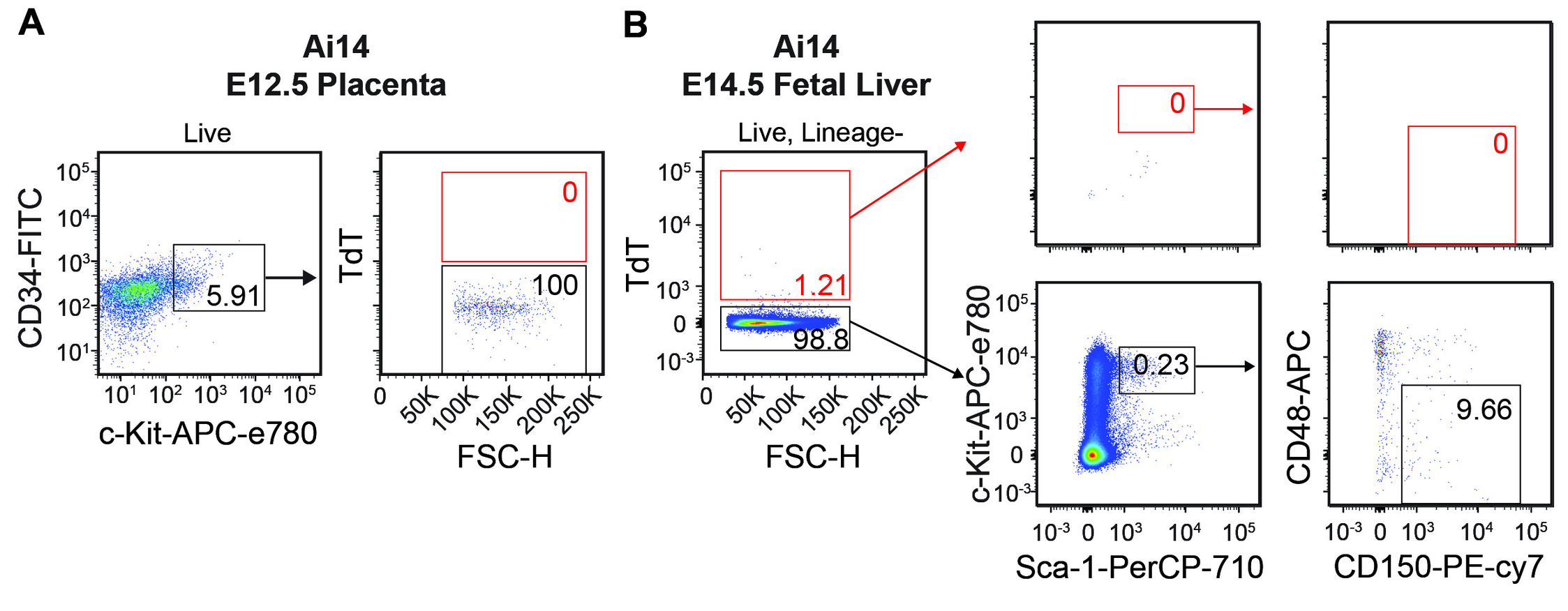

Supplement: S7 Fig — (A) FACS plot of E12.5 control Ai14 placenta for HSPCs. Red gate and text indicates TdT + cells. (B) FACS plot of HSPCs from E14.5 control Ai14 fetal livers. (TIF) [file pbio.3003003.s007.tif]

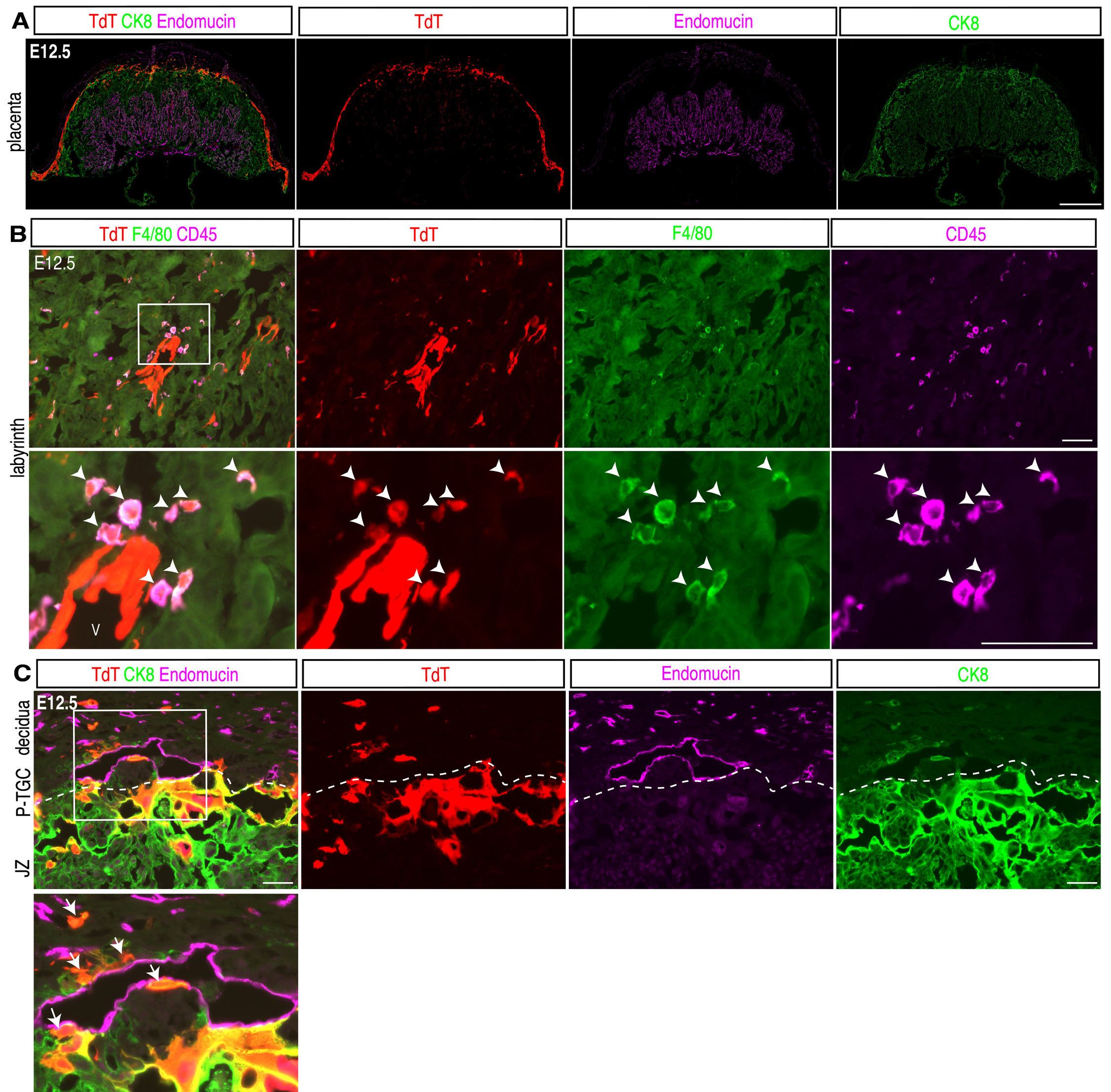

Supplement: S8 Fig — (A) Whole placenta immunostaining of TdT (red), CK8 (green) and Endomucin (magenta) of E12.5 Cdh5-CreERT2; Ai14 animals treated with 4-OHT at E7.5. (B) Immunostaining for TdT (red), F4/80 (green) and CD45 (magenta) on the placenta labyrinth area of E12.5 Cdh5-CreERT2; Ai14 animals. The lower panel shows the boxed region in the upper panel at higher magnification. White arrowheads indicate placenta fetal macrophages. (C) Immunostaining of TdT (red), CK8 (green) and Endomucin (green) on E12.5 Cdh5-CreERT2; Ai14 animals showing the boundary between decidua and junctional zone. White arrows indicate invasive trophoblast cells. Dotted lines indicate the separation between maternal and fetal sides of the placenta. The lower image shows the boxed region above at higher magnification. Scale bars: 500 μm (A), 100 μm (upper C), 50 μm (B, bottom C). (TIF) [file pbio.3003003.s008.tif]

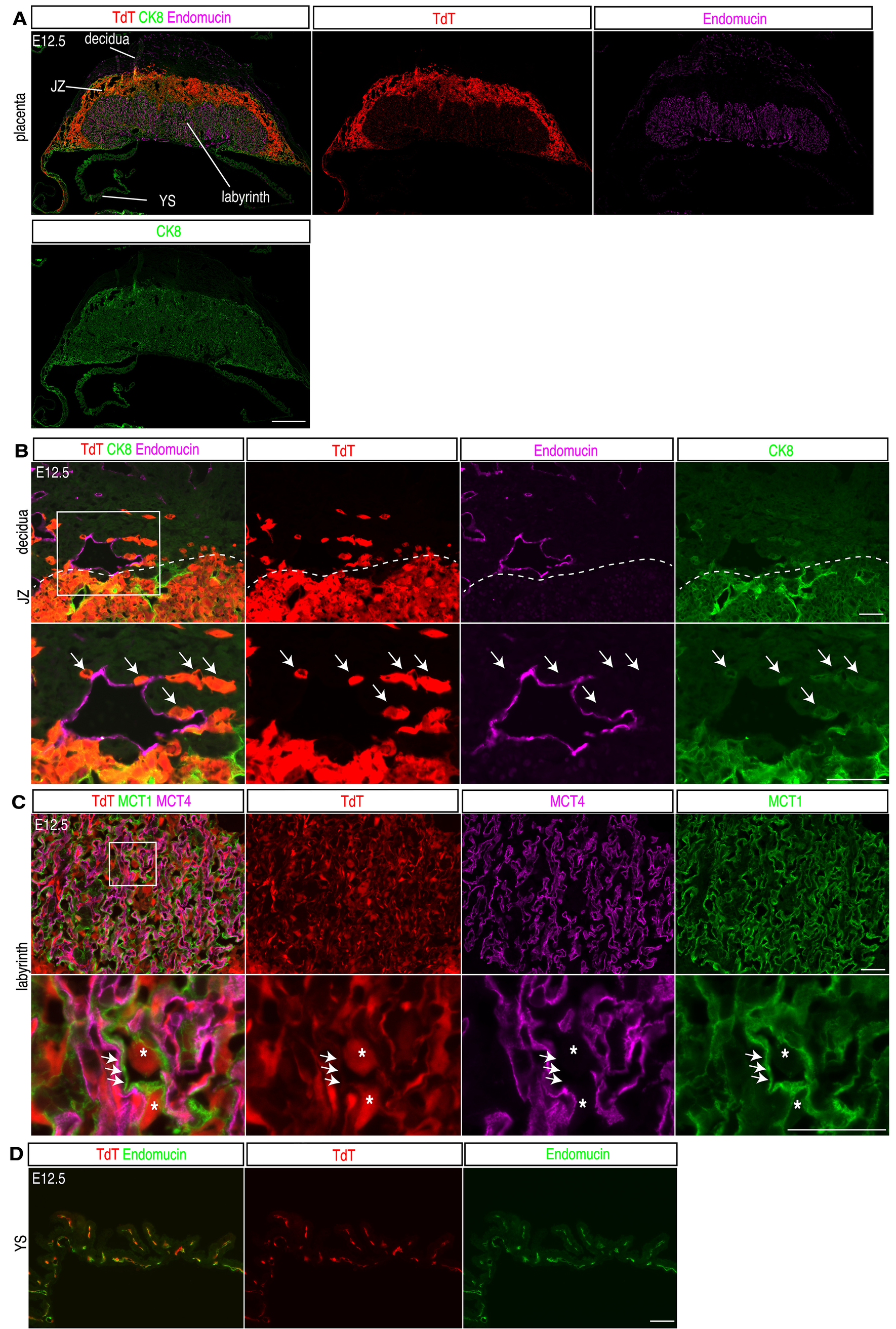

Supplement: S9 Fig — (A) Whole placenta immunostaining for TdT (red), CK8 (green) and Endomucin (magenta) of E12.5 Cdh5-CreERT2; Ai14 animals treated with 4-OHT at E10.5. (B) Immunostaining of TdT (red), CK8 (green) and Endomucin (magenta) on E12.5 Cdh5-CreERT2; Ai14 animals showing the boundary between decidua and junctional zone. White arrows indicate invasive trophoblast cells. Dotted lines indicate the separation between maternal and fetal sides of the placenta. The lower images show the boxed region in the panel above at higher magnification. (C) Immunostaining of TdT (red), MCT1 (green) and MCT4 (magenta) on E12.5 Cdh5-CreERT2; Ai14 animals showing the labyrinth area in the placenta. The stars indicate S-TGCs. The arrows indicate TdT + SynT-I cells. (D) Immunostaining of TdT (red) and Endomucin (green) on E12.5 Cdh5-CreERT2; Ai14 animals showing the yolk sac area. 500 μm (A), 100 μm (C, D), 50 μm (B). (TIF) [file pbio.3003003.s009.tif]

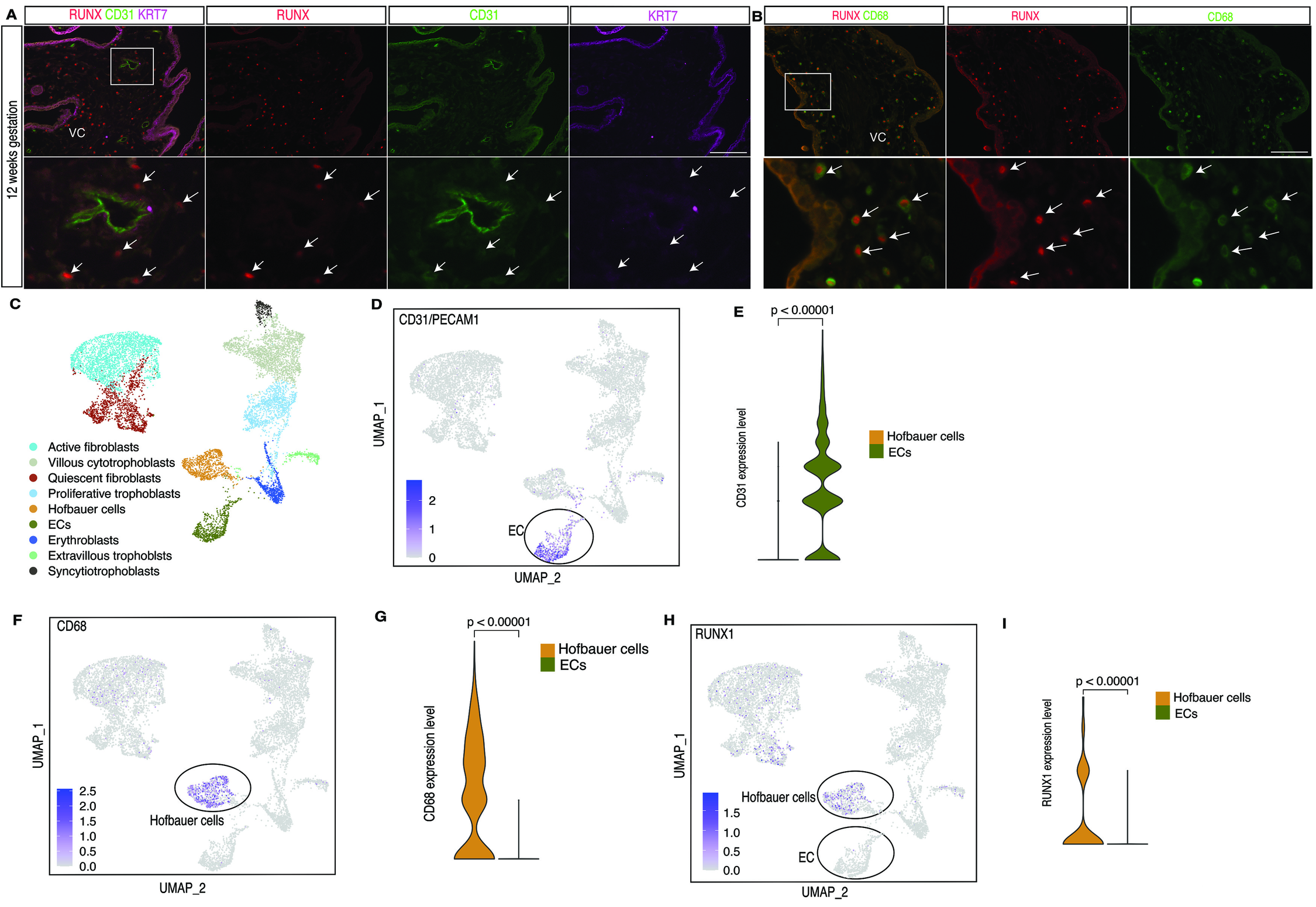

Supplement: S10 Fig — (A) Immunofluorescence staining for RUNX (red), CD31 (green) and KRT7 (magenta) on 12 week gestational age human placenta sections (3 sections for N = 1 placenta). Lower panels show enlarged view of the boxed regions in the upper panels. Arrows indicate RUNX + cells in the perivascular region of the villi. vc, villous cord. (B) Immunofluorescence staining for RUNX (red) and CD68 (green) on 12 week gestational age human placenta sections (3 sections for N = 1 placenta). Three bottom panels show enlarged view of the boxed region in three upper panels. Arrows indicate RUNX+CD68 + Hofbauer cells. (C) Plot showing cluster integration of scRNA-seq dataset from first trimester human placenta villi. (D) UMAP plot showing CD31/PECAM1 expression in villus ECs. The EC population is outlined. (E) Plot showing quantification of CD31 expression in Hofbauer cells and ECs. (F) UMAP plot showing CD68 expression in Hofbauer cell populations. Hofbauer cell populations are outlined. (G) Plot showing quantification of CD68 expression in Hofbauer cells and ECs. (H) UMAP plot showing RUNX1 expression in Hofbauer cells. (I) Plot showing no RUNX1 expression in ECs and significant RUNX1 expression in Hofbauer cells. P-values were shown based on unpaired, two-tailed Welch t test with unequal variances. (TIF) [file pbio.3003003.s010.tif]
